# Structural Insight into Archaic and Alternative Chaperone-Usher Pathways Reveals a Novel Mechanism of Pilus Biogenesis

**DOI:** 10.1371/journal.ppat.1005269

**Published:** 2015-11-20

**Authors:** Natalia Pakharukova, James A. Garnett, Minna Tuittila, Sari Paavilainen, Mamou Diallo, Yingqi Xu, Steve J. Matthews, Anton V. Zavialov

**Affiliations:** 1 Department of Chemistry, University of Turku, Turku, JBL, Arcanum, Turku, Finland; 2 Centre for Structural Biology, Department of Life Sciences, Imperial College London, South Kensington, London, United Kingdom; University of Washington, UNITED STATES

## Abstract

Gram-negative pathogens express fibrous adhesive organelles that mediate targeting to sites of infection. The major class of these organelles is assembled via the classical, alternative and archaic chaperone-usher pathways. Although non-classical systems share a wider phylogenetic distribution and are associated with a range of diseases, little is known about their assembly mechanisms. Here we report atomic-resolution insight into the structure and biogenesis of *Acinetobacter baumannii* Csu and *Escherichia coli* ECP biofilm-mediating pili. We show that the two non-classical systems are structurally related, but their assembly mechanism is strikingly different from the classical assembly pathway. Non-classical chaperones, unlike their classical counterparts, maintain subunits in a substantially disordered conformational state, akin to a molten globule. This is achieved by a unique binding mechanism involving the register-shifted donor strand complementation and a different subunit carboxylate anchor. The subunit lacks the classical pre-folded initiation site for donor strand exchange, suggesting that recognition of its exposed hydrophobic core starts the assembly process and provides fresh inspiration for the design of inhibitors targeting chaperone-usher systems.

## Author Summary

Gram-negative pathogens depend on fibrous adhesive organelles to attach to target tissues and establish infection. The major class of these organelles is assembled via the classical, alternative and archaic chaperone-usher (CU) pathways. CU pathways are recognized as promising new targets for the next generation of antibacterial drugs. The recently discovered archaic and alternative systems are of particular interest, as they are implicated in biofilm formation of antibiotic resistant pathogens, have a wider phylogenetic distribution and are associated with a broader range of diseases than the classical systems. Here, we report an atomic-resolution insight into the structure and assembly mechanism of two such biofilm-forming organelles assembled via the archaic and alternative pathways. We show that the archaic and alternative systems are structurally related, but their assembly mechanism is strikingly different from the classical assembly pathway. Whereas the classical chaperones deliver folded subunits to the usher assembly platform, non-classical chaperones apply a unique binding mechanism to maintain subunits in substantially unfolded state. The open subunit core allows for a new mode of strand replacement during polymerisation, and also represents an attractive target for the rational design of antimicrobials.

## Introduction

All gram-negative bacteria express fibrous adhesive organelles that mediate targeting to sites of infection. The major class of these adhesive pili (or fimbriae) is assembled via the classical, alternative and archaic chaperone-usher (CU) pathways [1]. CU pili are linear polymers made of subunits capable of either self-polymerisation or assembly with other subunits [2,3]. The CU fibre can possess rich binding properties [3–5], which facilitate binding to host cell receptors, as well as mediate biofilm formation through self-association [6] and interactions with abiotic surfaces [7].

The biogenesis of CU fibres requires a periplasmic chaperone and outer membrane assembly platform termed the usher [2]. Although these assembly proteins are conserved within the three CU pathway families, little sequence homology exists between the different CU pathways, which suggests distant phylogenetic relationships [1]. Among the three CU systems, the archaic (also termed σ) pathway assembles the largest class of pili [1]. Whereas the classical and alternate CU systems are restricted to β- and γ-proteobacteria, members of the archaic CU family are present in α-, β-, γ-, and δ-proteobacteria, whilst also in phyla *Cyanobacteria* and *Deinococcus*-*Thermus*. Furthermore, archaic systems are associated with bacteria that cause some of the most severe diseases in humans, animals, and plants [1]. Archaic Csu pili mediate the formation of *Acinetobacter baumannii* biofilms, which contribute to high rates of nosocomial infections [7]. This pilus is formed from four subunits, namely CsuA/B, CsuA, CsuB, and CsuE, and is assembled using the CsuC-CsuD chaperone-usher secretion machinery [7,8]. The alternative or α CU pathway is a highly divergent family with a wide phylogenetic distribution [1]. This pathway includes CFA/I-like fimbriae, which are the primary adhesins of human enterotoxigenic *Escherichia coli*, a major cause of mortality in young children from developing countries. The *E*. *coli* common pilus (ECP) also belongs to the alternative pathway and is associated with both disease-causing and commensal strains [9]. ECP is composed of the EcpA and EcpD subunits, which are assembled using two periplasmic chaperones, EcpB and EcpE, and the EcpC usher [10]. The classical CU pathways, namely β, γ, κ and π, are relatively conserved and they assemble a large variety of structures that are primary associated with the virulence of animal and human pathogens.

The classical systems have been studied for several decades and their biogenesis is now understood in exquisite detail. The periplasmic chaperones form a binary chaperone-subunit complex by occupying a hydrophobic cleft created by the absence of a β-strand from the subunit immunoglobulin (Ig) like fold, in a process known as donor strand complementation (DSC) [11,12]. Fibre subunits are subsequently assembled by donor strand exchange (DSE), in which the N-terminal extension from an incoming subunit displaces the chaperone via a "zip-in-zip-out" mechanism [13,14] and provides the necessary β-strand [14,15]. This process occurs at the entrance to the usher pore and is facilitated [16] by optimal positioning of the incoming chaperone-subunit complex by the usher [17].

Although archaic and alternative systems (grouped under the term ‘non-classical’) have a far wider phylogenetic distribution and are associated with a broader range of diseases than their classical equivalents, little is known regarding their precise assembly mechanisms. Recent structural analysis of the subunits from two alternative systems confirm that their biogenesis is governed by the general principles of DSC and DSE [6,18], although the lack of sequence similarity between chaperones suggests that the assembly process for the non-classical pathways could differ from the classical systems substantially. Here, we report atomic-resolution insight into the structure and biogenesis of *Acinetobacter baumannii* Csu and *Escherichia coli* ECP pili assembled via the archaic and alternative pathways, respectively, whilst also highlighting some important deviations from the classical assembly mechanism. The non-classical chaperones, unlike their classical counterparts, maintain subunits in a substantially unfolded state by utilising a register-shifted DSC and a distinct subunit C-terminal carboxylate anchor. The extreme dynamic nature of this chaperone-bound subunit arrangement allows for a more flexible mode of DSE initiation during polymerisation. Furthermore, this mechanistic distinction represents an attractive target for the rational design of new antimicrobials.

## Results

### CsuA/B is the major Csu pilus subunit and capable of self-polymerisation

**Fig 1:**
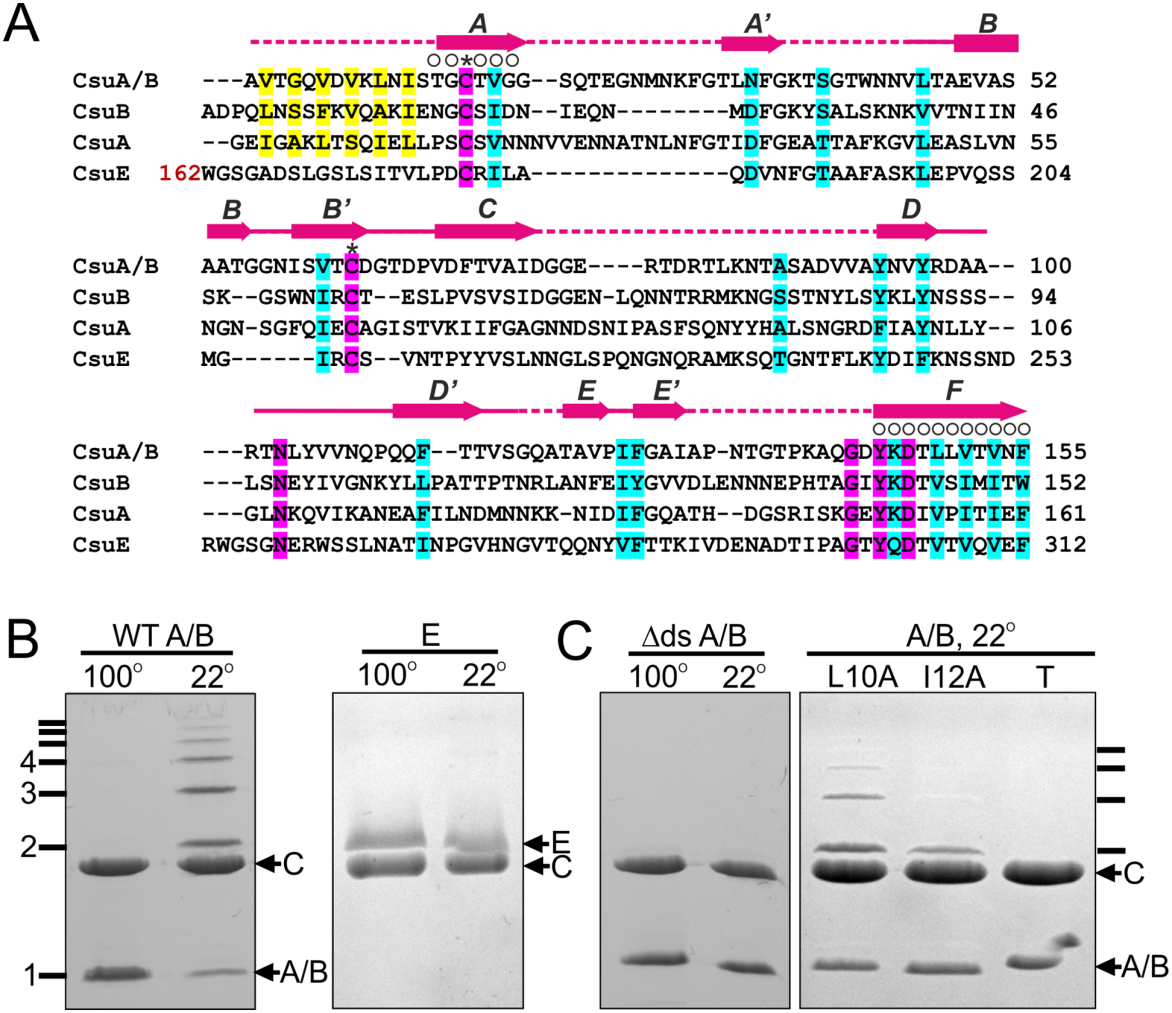
CsuA/B self-assembly depends on its N-terminal donor strand sequence. (**A**) Alignment of sequences of Csu pilin subunits A/B, A and B and the pilin domain of subunit CsuE. ClustalW was used to align the sequences. Residues are coded as follows: identical (pink shading); conserved character (cyan shading); pilin N-terminal residues proposed to take part in donor strand complementation in the pilus (yellow); involved in chaperone binding (circles above the residue); invariant for archaic pilin domain cysteines (stars above residues). Limits and nomenclature for secondary structure elements are shown above the sequence. Dashed line indicates unstructured sequences. Structural data were derived from the crystal structure of CsuC-CsuA/B complex (this study). (**B**) CsuA/B is capable of self-polymerisation and CsuE is not. CsuA/B and CsuE were co-expressed with His_6_-tagged CsuC in *E*. *coli*, co-purified from periplasmic extracts by Ni^2+^-affinity chromatography, and analysed by SDS-PAGE. Complexes were incubated at 22 or 100°C prior to the electrophoresis. (**C**) Identification of polymerisation sequence in CsuA/B. CsuA/B mutant with 12 N-terminal amino acid residues replaced by a His_6_-tag (Δds) was co-expressed with wild type CsuC. CsuA/B point mutants Leu10→Ala and Ile12→Ala and a triple mutant (T) with Val8, Leu10 and Ile12 substituted to alanines were co-expressed with His_6_-tagged CsuC. CsuC-CsuA/B complexes were purified and analysed by SDS-PAGE as in (B).

The *csu* gene cluster encodes four different pilus subunits (CsuA/B, CsuA, CsuB, CsuE) (Fig 1A). Based on size, positioning within the operon and levels of expression [7,8], we reasoned that CsuA/B is the major shaft-forming subunit and CsuE is a tip subunit. In this scenario, CsuA/B should be capable of polymerisation whilst CsuE, the proposed tip subunit, should not. To verify this, we purified the subunits after over-expression in *E*. *coli* and examined their ability to polymerize *in vitro*. In the absence of the chaperone, only low levels of expression were detected, however upon co-expression with CsuC, these levels dramatically increased. Moreover, the subunits were successfully co-purified via the His-tagged CsuC (CsuC-His_6_) by Ni^2+^-affinity chromatography, suggesting that they form stable chaperone complexes (Fig 1B).

SDS-PAGE of purified CsuC-CsuA/B complexes after incubation at room temperature revealed a ladder of bands with sizes corresponding to a dimer, trimer, tetramer and larger multimers of the CsuA/B subunit. In contrast, electrophoresis of the CsuC-CsuE complex resulted in a single band of the CsuE monomer (Fig 1B). Boiling the CsuC-CsuA/B sample disrupted the ordered CsuA/B aggregation, resulting in a single band for CsuA/B (Fig 1B). This behaviour has been previously observed for major subunits from classical CU systems [19] and together this confirms that CsuA/B is the major subunit capable of spontaneous polymerisation in presence of the chaperone. Although subunits CsuA and CsuB are not expressed as efficiently as CsuA/B [8], they have similar size and might also be capable of polymerisation, serving as either adaptors or forming finer shaft structures.

### CsuA/B utilizes its N-terminal donor strand for polymerisation

Upon closer inspection of N-terminal sequences of CsuA/B, CsuA and CsuB, a clear pattern of alternating hydrophilic-hydrophobic residues is observed, characteristic for N-terminal donor strands of classical pilins (Fig 1A). To test whether CsuA/B polymerizes via DSC, we substituted the first 12 residues with a His_6_-tag (His_6_-CsuA/B) and co-expressed it with CsuC. Analysis of the co-purified complex by SDS-PAGE prior to and after boiling show no ladder of CsuA/B polymers, indicating that this N-terminal segment is responsible for assembly (Fig 1C). To confirm that the sequence forms a donor strand we prepared single alanine substitution mutants of the two largest hydrophobic residues, Leu10 and Ile12, in a tagless CsuA/B construct and co-expressed these with CsuC-His_6_. SDS PAGE analysis revealed a significant reduction of high molecular mass polymers, particularly for the Ile12Ala mutant (Fig 1C). Moreover, simultaneous mutation of three hydrophobic residues (Val8, Leu10 and Ile12) abolished CsuA/B polymerisation completely. These results provide convincing evidence that the assembly of archaic pili is based on DSC.

### The CsuC chaperone adopts the canonical tandem Ig fold

**Fig 2:**
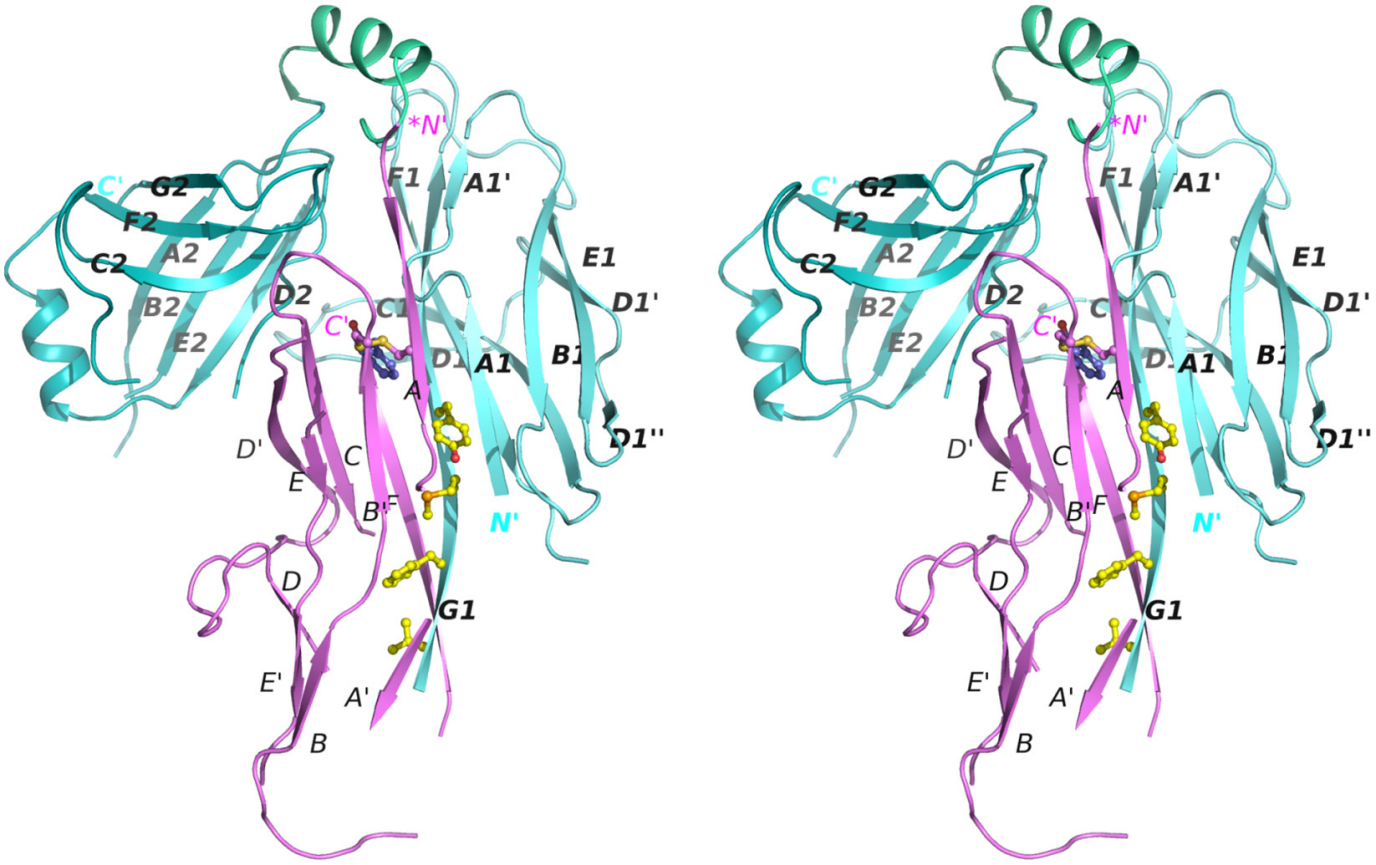
Stereo diagram of the crystal structure of the CsuC:CsuA/B complex. CsuC and CsuA/B are painted in cyan and magenta, respectively. Donor residues (Val110, Phe112, Met114, Tyr116) are shown as balls-and-sticks. N and C termini and β-strands are labelled. The asterisk in the *N´ label indicates that the N-terminal donor sequence of CsuA/B has been replaced by a His-tag.

**Fig 3:**
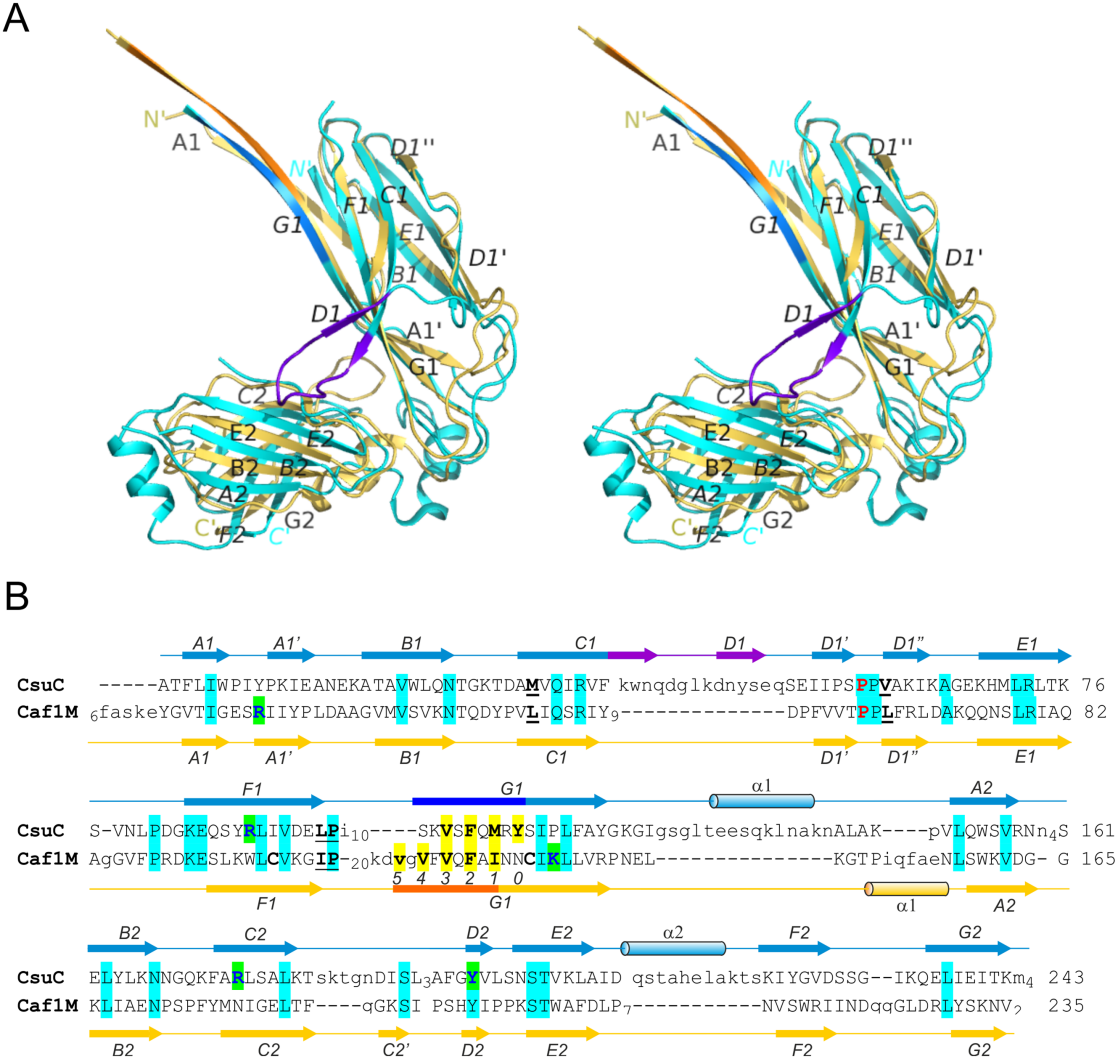
Structural comparison of archaic and classical chaperones. (**A**) Superposition of CsuC and classical chaperone Caf1M (stereo diagram). CsuC and Caf1M are cyan and yellow, respectively, except donor strand segments, which are blue and orange in CsuC and Caf1M, respectively, and the C_1_-D_1_ hairpin in CsuC, which is violet. (**B**) Structural alignment of CsuC and Caf1M. Structurally equivalent and non-equivalent residues are shown in upper and lower cases, respectively. The number of residues in unstructured segments is indicated. Amino acid identities are indicated by background shading in cyan. Donor and subunit carboxylate anchoring residues are shown on yellow and green backgrounds, respectively. The usher-binding residues in Caf1M and corresponding residues in CsuC are underlined. The proline, which is invariant for the entire chaperone superfamily (classical, archaic, and alternative chaperones) is shown in red. The disulphide bond-linked cysteines in Caf1M are shown in bold. Positions of donor residues in acceptor pockets are indicated (0–5). Secondary structure (cylinder, α-helix; arrow, β-strand; line, coil) is shown above and below the amino acid sequence of CsuC and Caf1M, respectively.

To gain insight into the structure and assembly of archaic CU pili, we determined the crystal structure of a CsuC-CsuA/B pre-assembly complex, composed of His_6_-CsuA/B and CsuC (to avoid polymerisation). Crystals were readily obtained in spacegroup P6_4_22 and the structure was solved using Se-SAD phasing to a resolution of 2.4 Å (Fig 2). The CsuC chaperone has a canonical Ig-like fold with two 7-stranded β sandwich domains (D1 and D2) oriented at ∼90° angle (Figs 2 and 3A). Despite the lack of sequence similarity, comparison of CsuC and classical chaperones with known structures revealed a significant similarity in D1. For example, 100 equivalent D1 Cα atoms of CsuC and the *Yesinia pestis* Caf1M chaperone superimpose with RMSD of 1.9 Å (Z-score = 12.8, S2 Table) (Fig 3A). The largest structural differences occur at the edges of the β sandwich domain. CsuC has an additional β-strand D_1_ (Fig 3A and 3B). The C_1_-D_1_ hairpin protrudes from domain D1 towards domain D2, closing the entrance to the inter-domain cleft. This additional sequence is present in all archaic chaperones (S1 Fig), suggesting that this blockade of inter-domain cleft plays an important functional role. Domain D2 is less similar to the equivalent domain in classical chaperones (Z-score = 5.9, S2 Table). The principal difference occurs in the position of β-sheet D_2_C_2_F_2_G_2_ (Fig 3A); in Caf1M this is rotated with respect to β-sheet A_2_B_2_E_2_ by 35–50°, where as in CsuC this is 60–85°. The nearly orthogonal packing of β-sheets renders the β-barrel in CsuC more open than for Caf1M, although the β-barrel is covered by an additional helix from the E_2_-F_2_ loop (helix 2).

### The CsuC chaperone maintains the CsuA/B in a partially ordered state

**Fig 4:**
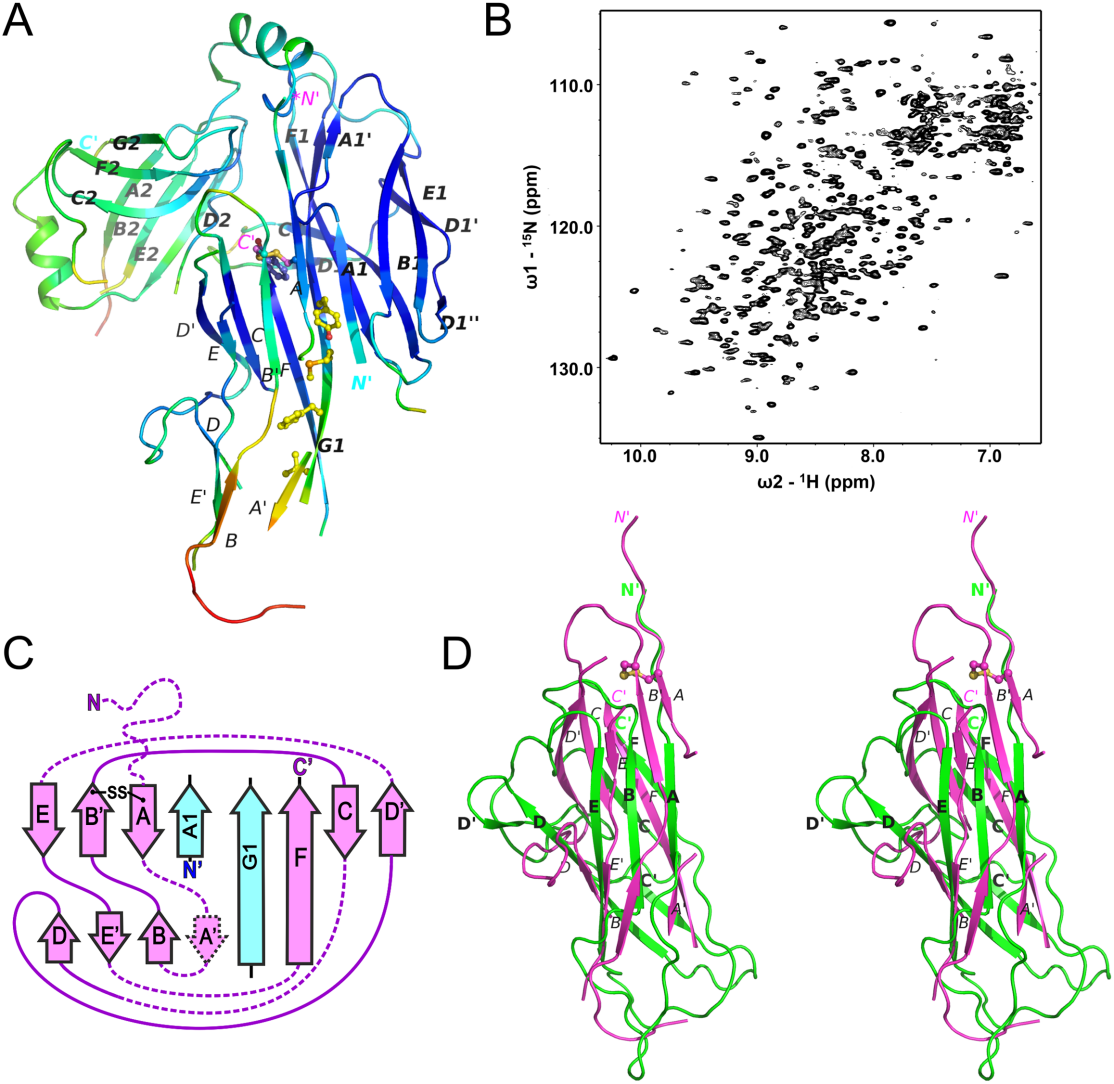
Chaperone-bound CsuA/B is partially folded. (**A**) Diagram of the crystal structure of the CsuC:CsuA/B complex coloured by B-factor of Cα atoms with the colour ranging from blue to red and corresponds to a B-factor range from 12.39 to 113.65 Å^2^. (**B**) Solution NMR analysis of the CsuC-CsuA/B complex. ^1^H-^15^N TROSY HSQC spectrum of CsuC-CsuA/B. (**C**) Topology diagrams of CsuA/B. Arrows indicate β strands. CsuA/B is magenta; CsuC A_1_ and G_1_ β strands are cyan. The wavy dashed line at the N-terminus of CsuA/B indicates the N-terminal polymerisation sequence. Other dashed lines indicate unstructured sequences in the core structure. (**D**) Superposition of CsuA/B (magenta) and Caf1 (green) (stereo diagram). The disulphide bridge in CsuA/B is shown as balls-on-sticks. Note that a large part of CsuA/B is unstructured.

Whilst the refinement statistics for the structure of the CsuC-CsuA/B complex are good (S1 Table), approximately 40% of the CsuA/B sequence was not evident in electron density maps, whilst another 7% has very poor electron density (Cα atom B-factors higher than 80 A^2^). Furthermore, this is also reflected in the gradual increase in B-factors for CsuA/B outside of the chaperone interface (Fig 4A). To provide further insight into the structural heterogeneity in CsuA/B within the CsuC-bound complex, we prepared a ^15^N-labelled CsuC-CsuA/B sample and acquired a ^1^H-^15^N TROSY NMR spectrum (Fig 4B). While the spectrum shows good chemical shift dispersion consistent with a significant ordered structure, a larger than expected distribution of amide line widths is observed and ~15% of the expected non-proline resonances are absent from the spectrum. This strongly implies that within the context of a pre-assembly complex a large portion, presumably within CsuA/B, displays dynamic conformational exchange on an intermediate timescale. Although our data does not rule out that this large region adopts an alternative conformational state, it more likely that it exists in many different states and exchanges between them. Additional ordered conformational states would manifest as multiple NMR resonances for the same residue. We observe no evidence for this within the NMR spectrum for the chaperone-subunit complex. In fact, the NMR spectral properties more akin to that for a molten globule and is consistent with our crystallographic data, in which no clear electron density was resolved for almost half the CsuA/B sequence.

The remaining structure of the CsuA/B subunit reveals a double β-sheet sandwich comprising strands A, B, and E (β-sheet 1) and C and F (β-sheet 2) and β-strand D, which switches between the sheets (Figs 2 and 4C). The three strands of β-sheet 1 (A, B, and E) are interrupted in the middle by aperiodic regions. Conserved cysteines 16 and 62 form a disulphide bridge linking the beginning of β-strand A with the end of β-strand B´. The N-terminal sequence up to Ser13 is disordered and the large unstructured sequences are located between β-strands A and A´, A´ and B, C and D, D´ and E, and E´ and F.

The most striking structural difference between CsuA/B and classical subunits is in the degree to which subunits are folded when in complex with the chaperone. In the majority of available structures of preassembly complexes from classical systems, the entire sequence (99–100%) of the chaperone-bound subunit (except for the N-terminal extension) is highly ordered (S3 Table, e.g. *Yersinia pestis* capsular subunit Caf1 in complex with Caf1M shown in Fig 4D). In contrast to the classical systems, nearly half of CsuA/B is disordered or displays very poor electron density.

Nevertheless, the ordered part of CsuA/B provides sufficient information to conclude that, as with classical systems this pilin has the incomplete Ig-like fold in a six-stranded β-sandwich, in which the absent 7^th^ strand (G) leaves a large hydrophobic cleft. The ordered part of CsuA/B shows limited structural similarity to Caf1 with a Z-score of 3.7 (Fig 4D).

### The donor strand register in the CsuC-CsuA/B complex is shifted relative to that in classical chaperone-subunit complexes

CsuA/B interacts predominantly with D1 of the chaperone CsuC via edge strands to form a closed “super-barrel” with a common core. The chaperone A_1_ and G_1_ strands are hydrogen bonded to the subunit A and F strands, respectively. Four large hydrophobic residues from the chaperone G_1_ strand (Val110, Phe112, Met114, Tyr116) are donated to the subunit to compensate for the missing G strand. In addition, strand A_1_ provides several hydrophobic residues stabilising the super-barrel (S2 Fig).

**Fig 5:**
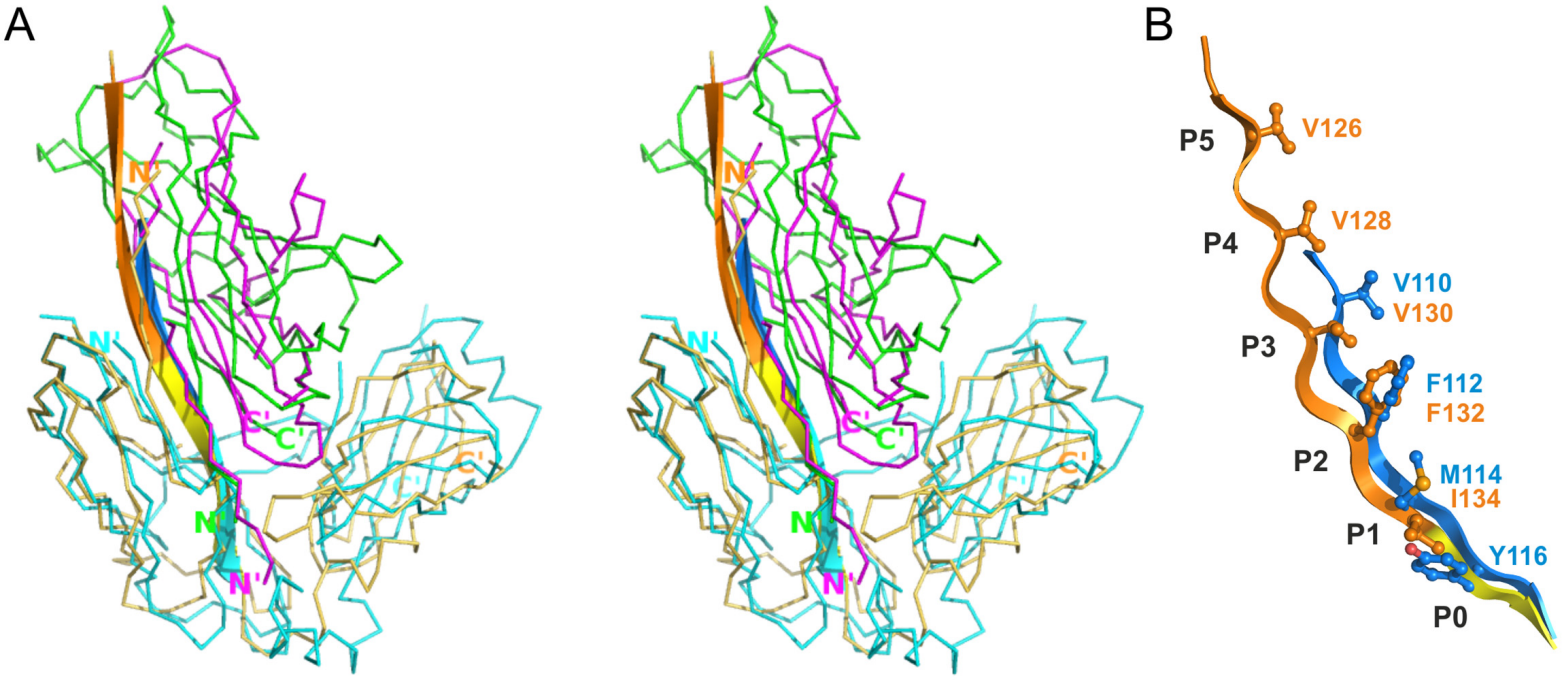
Differences in the register of donor residues and position of the subunit in CsuC:CsuA/B and complexes from classical systems. (**A**) Superposition of the archaic CsuC:CsuA/B and classical Caf1M:Caf1 chaperone-subunit complexes (stereo diagram). CsuC, CsuA/B, Caf1M, and Caf1 are painted in cyan, magenta, yellow and green, respectively. Strand G_1_ is shown as cartoon diagram and donor strand segments in CsuC and Caf1M are shown in blue and orange, respectively, as in Fig 3A and panel B. (**B**) Detailed alignment of G_1_ donor strands in CsuC and Caf1M. Hydrophobic donor residues in positions P0-5 are shown in balls-and-sticks and labelled.

Superposition of CsuC-CsuA/B and Caf1M-Caf1 complexes revealed that CsuA/B is situated closer to the chaperone D2 than Caf1 (Fig 5A). To explore this global difference, we compared the position of donor strand residues in CsuC and Caf1M. In the classical chaperones the donor strand motif can vary in length (from 3 to 5 hydrophobic donor residues as in Caf1M), but it starts from the same position 1 in the classical donor strand register, corresponding to Ile134 in Caf1M. However, in CsuC, the donor strand motif is shifted towards the C-terminal end of β-strand G_1_ by two residues (or one donor residue). It starts with the highly conserved Tyr116 and corresponds to position 0 using the classical donor strand register (Figs 5B, 3B, and S1). The subunit and donor residue motif in the chaperone are shifted in the same direction along strand G_1_, arguing that the donor strand determines the position of the subunit.

### CsuC utilises a unique C-terminal carboxylate binding mechanism

**Fig 6:**
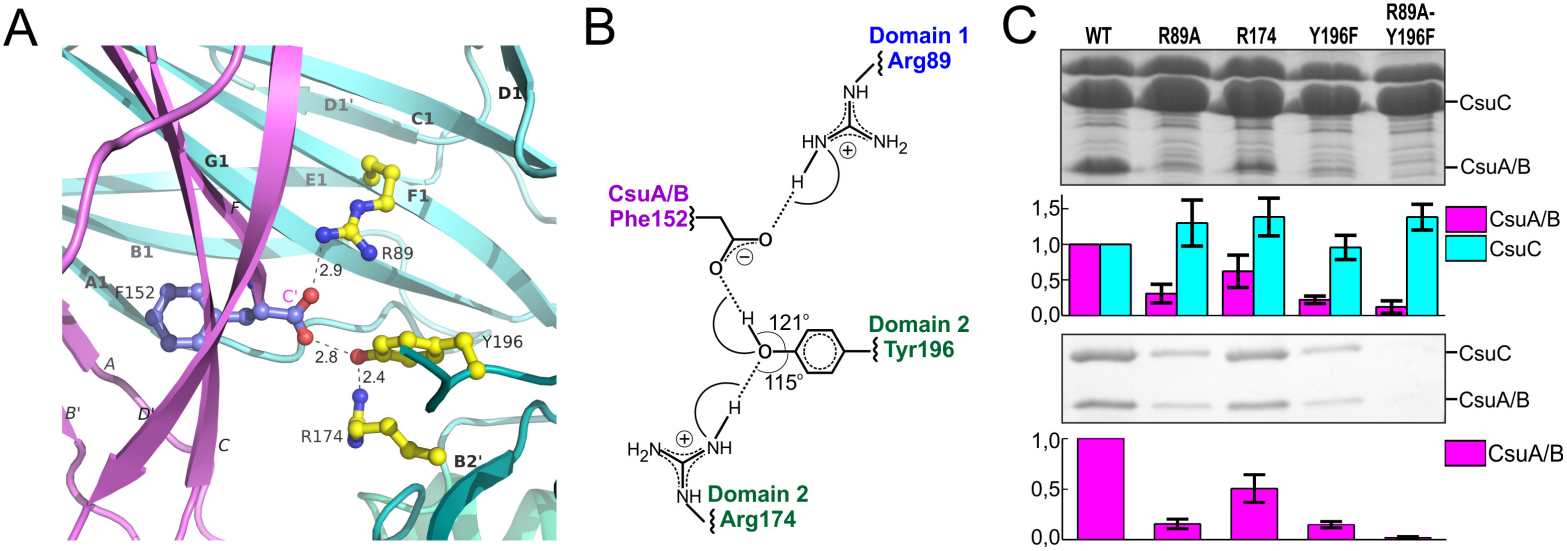
Network of ionic-hydrogen bonds anchoring the C-terminal carboxylate of CsuA/B in the inter-domain cleft of CsuC is essential for the complex formation. (**A**) Close-up of the CsuC:CsuA/B structure, demonstrating interactions between C-terminal carboxylate of CsuA/B and CsuC (cartoon diagram). C-terminal Phe152 in CsuA/B and Arg89, Tyr196, and Arg174 in CsuC are shown as balls-and-sticks. Hydrogen bonds are shown with dashes and their length is indicated. The color-coding is same as in Fig 2. (**B**) Interaction map between the C-terminal carboxylate and CsuC. (**C**) Contribution of CsuA/B C-terminal carboxylate binding residues to CsuC-CsuA/B association. CsuA/B was co-expressed with wild type and mutated CsuC in *E*. *coli* cells, followed by the periplasm extraction and Ni^2+^-column fractionation. Samples of periplasmic extracts and purified complexes were separated by a SDS-polyacrylamide electrophoresis and optical density of bands of CsuA/B and CsuC was measured and integrated. Segments of gels of periplasmic extracts (top) and purified complexes (bottom) show bands of CsuA/B and CsuC. Integrated optical density (IOD) of the bands normalized against wild type values is shown on bar plots. The results are representative of three independent experiments.

Strand F is one residue shorter in CsuA/B than in classical subunits and the C-terminal Phe152 is situated in the centre of the complex, within the interdomain cleft of the chaperone (Fig 2A). The C-terminal carboxylate of Phe152 interacts with two highly conserved residues of CsuC (Fig 6A and 6B). One oxygen atom of the carboxylate forms an ionic interaction with invariant Arg89 (S1 Fig) in β-strand F of D1. The other oxygen atom of the carboxylate is hydrogen bonded to the hydroxyl group of the highly conserved Tyr196 from β-strand F of D2. Interestingly, the hydroxyl group of Tyr196 is also hydrogen bonded to the conserved Arg174. This basic residue serves as an acceptor of electrons, strengthening the hydrogen bond between the hydroxyl group of Tyr196 and C-terminal carboxylate (Fig 6B); therefore, Arg174 also contributes as a part of the carboxylate anchoring mechanism.

To study the contribution of this network to the subunit binding, we constructed point mutants of CsuC, namely Arg89Ala, Arg174Ala, and Tyr196Phe. Wild type CsuC and the mutant versions were co-expressed with His_6_-CsuA/B and assessed with Ni^2+^-affinity pulldowns (Fig 6C). Arg89Ala and Tyr196Phe mutations reduced the subunit recovery equally, suggesting that both ionic (carboxylate-Arg89) and hydrogen (carboxylate-hydroxyl group of Tyr196) bonds contribute to complex formation. Furthermore, combining these mutations led to near zero recovery of the subunit. The Arg174Ala mutation had a measureable effect on periplasmic levels of CsuA/B, but was less dramatic and supports a secondary role for Arg174 in positioning the carboxylate-Tyr196. Subunit C-terminal carboxylate binding in archaic chaperones is notably different from that of the classical chaperones. Classical chaperones bind the carboxylate via two highly conserved basic residues (Arg20 and Lys139 in Caf1M), which have no analogues in archaic chaperones. Furthermore, these residues are located in D1, whereas in archaic systems only one anchoring residue is provided by D1 and two are located in D2 (Fig 3B).

### Chaperones from archaic and alternative systems are structurally related

Subunit polymerisation within the alternative assembly system, ECP, cannot be explained fully by the classical CU mechanism. This is because it is necessary to insert a large tryptophan side chain from the middle of the EcpA N-terminal extension deeply within the core of an adjacent subunit during assembly [6]. Our observations that archaic chaperones maintain their subunits in a partially folded preassembly state via a distinct binding mode, led us to suggest that alternative systems could employ a similar mechanism. Furthermore, archaic and alternative CU assembly systems pre-date the evolution of the classical pathways, suggesting that they share similar non-classical CU features.

**Fig 7:**
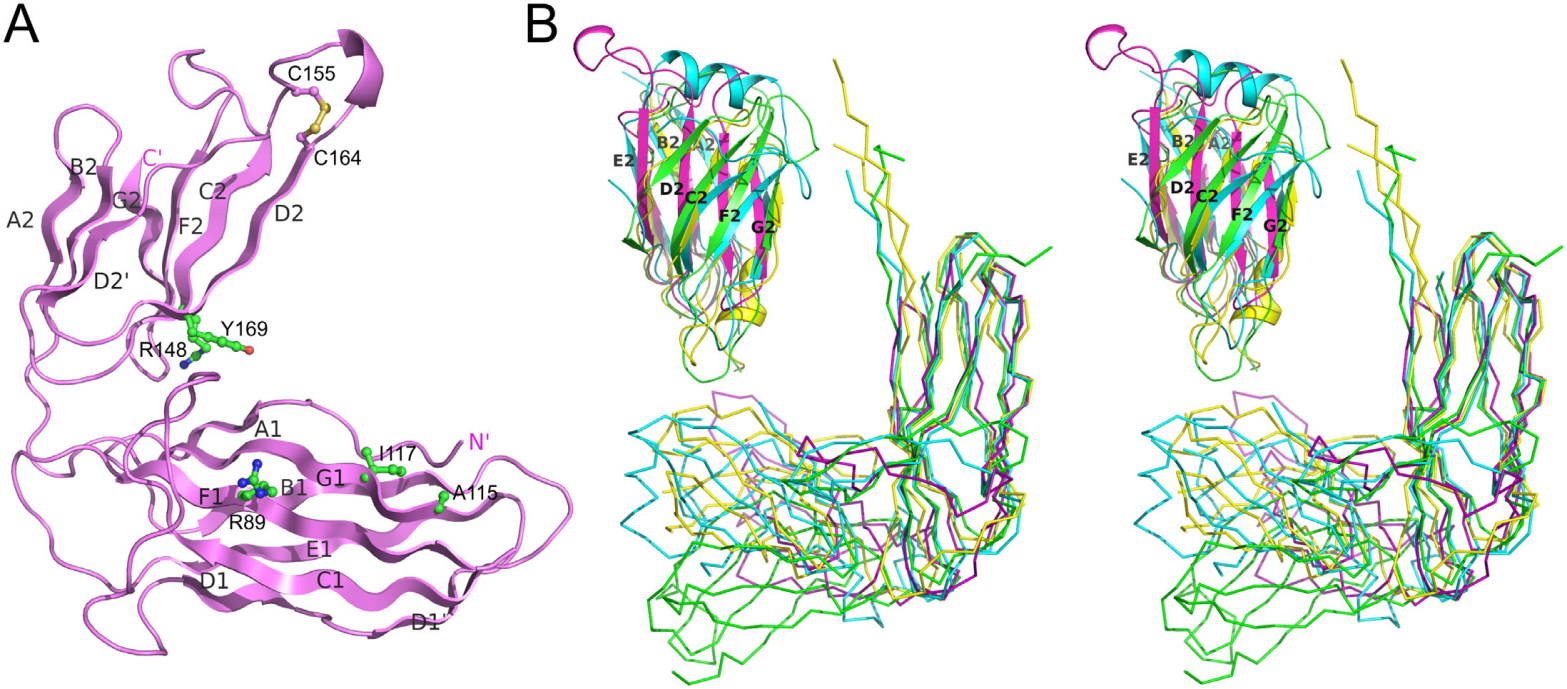
Structural comparison of alternative, archaic, and classical chaperones. (**A**) Crystal structure of EcpB (cartoon diagram). Donor, subunit C-terminal carboxylate anchoring, and cysteine residues are shown as balls-and-sticks. (**B**) Superposition of subunit-bound conformations of classical Caf1M (yellow) and archaic CsuC (cyan) chaperones and subunit-free alternative EcpB (purple) and CfaA (green) chaperones by minimizing the distance between corresponding Cα atoms of domain 1 (ribbon diagram) and superposition of C-terminal domains of these chaperones (cartoon diagram). The same color-coding is used below in Fig 8.

To test this we first demonstrated an interaction between the major pilin subunit EcpA and its cognate chaperone, presumed to be EcpB. We co-expressed EcpA (with a Trp11Ala mutation to abrogate self-polymerisation: EcpA^W11A^) in the *E*. *coli* periplasm with His-tagged EcpB (EcpB-His_6_), followed by purification via Ni^2+^-affinity chromatography. Subsequent gel filtration and SDS page analysis revealed that a tight complex is formed between EcpB and EcpA with a 1:1 stoichiometry (S3 Fig) and this was also confirmed by subsequent NMR analyses (see later). We next crystallized free EcpB and determined its structure using I-SIRAS phasing to 2.4 Å resolution (S1 Table). EcpB consists of the two characteristic Ig-like domains as seen in all other chaperones and has an overall boomerang-like shape (Fig 7A). Surprisingly, it superimposes poorly with any subunit free and subunit bound chaperone structures solved thus far (S2 Table). However, when we compared the individual domains, considerable structural similarity could be identified, particularly for D1. As expected, the superposition revealed that EcpB is structurally more related to CsuC (Z-score = 14.2) than classical chaperones (e.g. Caf1M, Z-score = 12.0) (S2 Table). No topological differences were found in D1 between EcpB and CsuC, but both possess an additional strand D_1_ that is absent in the classical chaperones. Less similarity is observed in D2 between EcpB, CsuC and the classical Caf1M (Z-scores of 3.0–3.1, S2 Table). Whereas the β-sheet packing in D2 in subunit-bound CsuC is almost orthogonal, in EcpB, the two β-sheets pack nearly parallel to each other, at an angle of just 15–30°. Domain 2 of EcpB does not possess helix 1, common in classical chaperones, or helices 1 and 2, characteristic for archaic chaperones (Figs 3, 7, and S1). Instead, it has a short helix located in a long loop between β-strands C_2_ and D_2_. The C_2_-D_2_ loop is flanked with a pair of conserved cysteines (170 and 179), which form a disulphide bond (S1 Fig) that is not seen in classical chaperones.

The alternative chaperones can be grouped into two subfamilies (S1 and S4 Figs). One group have significant similarity to EcpB, whereas the other group includes CfaA, which are involved in assembly of class 5 fimbriae in enterotoxigenic *E*. *coli* and *Yersinia pestis*. The crystal structure of chaperone CfaA has been recently determined [20], providing an opportunity to compare chaperones between these subfamilies (Fig 7B). Expectedly, superposition of N-terminal domains in EcpB and CfaA shows significant structural similarity (Z-score of 12.7, S2 Table). Surprisingly, structural similarity between C-terminal domains is low (Z-score = 5.4, S2 Table). The angle between the β-sheets in CfaA is more similar to classical chaperones and considerably smaller than in EcpB. EcpB and CfaA also display very large differences in the relative domain orientation; when superimposed over D1 (Fig 7B) the C-terminal ends are separated by over 30 Å.

### Archaic and alternative chaperones utilise similar modes of subunit binding

**Fig 8:**
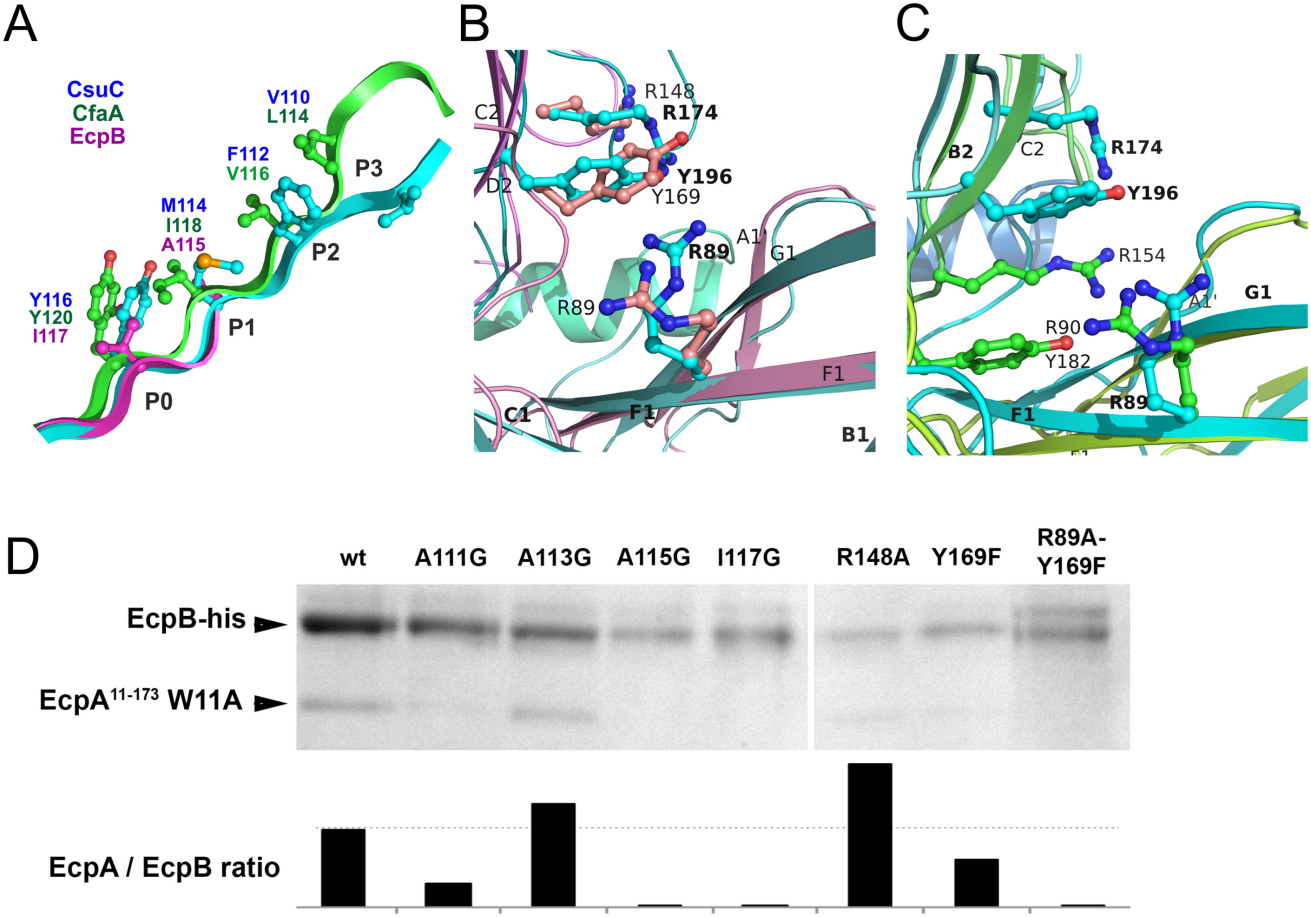
Both archaic and alternative chaperones bind to subunits by using register-shifted donor strand complementation and two-domain carboxylate anchor. (**A**) Detailed alignment of G_1_ donor strands for CsuC, EcpB, and CfaA. Note that position P0 is occupied by donor residues in all three chaperones. (**B** and **C**) Chaperones from the archaic and alternative pathways employ a similar subunit C-terminal carboxylate anchoring mechanism. Domains 1 and 2 of EcpB (B) and CfaA (C) were superimposed individually with the corresponding domains of the CsuC molecule to model their ‘subunit bound’ conformations. Arg89, Tyr196, and Arg174 in CsuC, which anchor the C-terminal carboxylate of CsuA/B and corresponding residues in EcpB and CfaA are shown as balls-and-sticks. (**D**) Identification of subunit binding residues in EcpB by mutagenesis. Residues in EcpB predicted to be involved in binding the C-terminal carboxylate (Arg89, Arg148, Tyr169) or lining the donor strand groove of EcpA (Ala111, Ala113, Ala115, and Ile117) were mutated in EcpB. An EcpA^17-173^ W11A mutant (defective in polymerisation) was co-expressed with wild type and mutated EcpB-his in *E*. *coli* BL21 (DE3) cells, followed by periplasm extraction and Ni^2+^-column fractionation. Samples of purified complexes were separated by SDS-polyacrylamide electrophoresis and optical density of bands was measured and integrated. Segments of gels for purified complexes (top) show bands of EcpA and EcpB, whilst (bottom) the integrated optical density for bands (represented as a ratio of EcpA/EcpB are shown on bar plots. As EcpB is tagged, any ratio below that of wild type (dotted line) suggests a less stable complex.

A detailed comparison of CsuC, EcpB and CfaA reveals that, as in CsuC, the donor strand motifs are shifted towards the C-terminus of β-strand G_1_ relative to the classical chaperones (Fig 8A). Large residues, Ile117 in EcpB and Tyr120 in CfaA, occupy position P0. The other positions (P1-3) in EcpB are occupied by alanines of which only one is structured in the subunit-free chaperone structure (S1 Fig). To confirm the role of the P0-3 residues in DSC, we replaced each with glycine and examined whether these mutants bind to the EcpA subunit. Mutant or wild type EcpB was co-expressed in *E*. *coli* with EcpA^W11A^, purified by Ni^2+^-affinity chromatography from the periplasm and analysed by SDS PAGE (Figs 8D and S5). Although Ala113Gly had little effect on complex formation, mutations Ala111Gly, Ala115Gly and Ile117Gly practically abolished capture of the subunit, suggesting that DSC is disrupted. The lack of an effect for Ala113Gly is not surprising as we predict that Ala113 occupies the P2 pocket of EcpA. This is where Trp11 is buried during pilus formation (S6 Fig) and we envisage that this pocket should be partially open within the EcpB-EcpA complex to enable the introduction of the large tryptophan side chain.

Archaic and alternative chaperones share two highly conserved residues (S1 Fig): proline (Pro59 in CsuC and Pro60 in EcpB) and arginine (Arg89 in CsuC and EcpB). The proline is the only invariant residue for the entire CU super-family (Fig 3B) [21]. It introduces a kink in β-strand D_1_ switching it between the β-sheets of D1 (S7 Fig). The highly conserved arginine is only present in archaic and alternative chaperones, representing the most characteristic sequence feature of these non-classical systems. Superposition of D1 from CsuC, EcpB, and CfaA reveals that the arginine is located in the same position in all three structures (Fig 8B and 8C). Since Arg89 in CsuC is essential for the binding of the C-terminal carboxylate of CsuA/B, we assumed that the corresponding residue in the alternative chaperones is involved in binding the subunit C-terminus. Furthermore, two other residues of CsuC implicated in binding (Tyr196 and Arg174) superimpose well with identical residues in EcpB (Tyr169 and Arg148, respectively), suggesting that these residues contribute to anchoring of the subunit C-terminal carboxylate in a similar manner. In CfaA, a similar pair of residues (Tyr182 and Arg154) is located deeper within the inter-domain cleft, due to a two-residue shift of the tyrosine and arginine towards the termini (Fig 8C). This shift is a characteristic feature of CfaA-like chaperones (S1 Fig).

To examine the role of Arg89, Tyr169, and Arg148 in EcpB in the interaction with EcpA, we created Arg148Ala and Tyr169Phe mutants, and also a double Arg89Ala, Tyr169Phe mutation. We next analysed their ability to recover EcpA in the *E*. *coli* periplasm (Figs 8D and S5). As for CsuC, mutation of Tyr169 in EcpB dramatically decreased its chaperone function and when both Arg89Ala and Tyr169Phe mutations are present, essentially no subunit can be recovered. Although mutation of Arg148 did not reduce the subunit-binding capability of EcpB significantly, our mutational data suggest that archaic and alternative chaperones share the same anchoring mode of subunit C-terminal carboxylate.

### EcpA is also partially ordered in complex with its cognate chaperone

**Fig 9:**
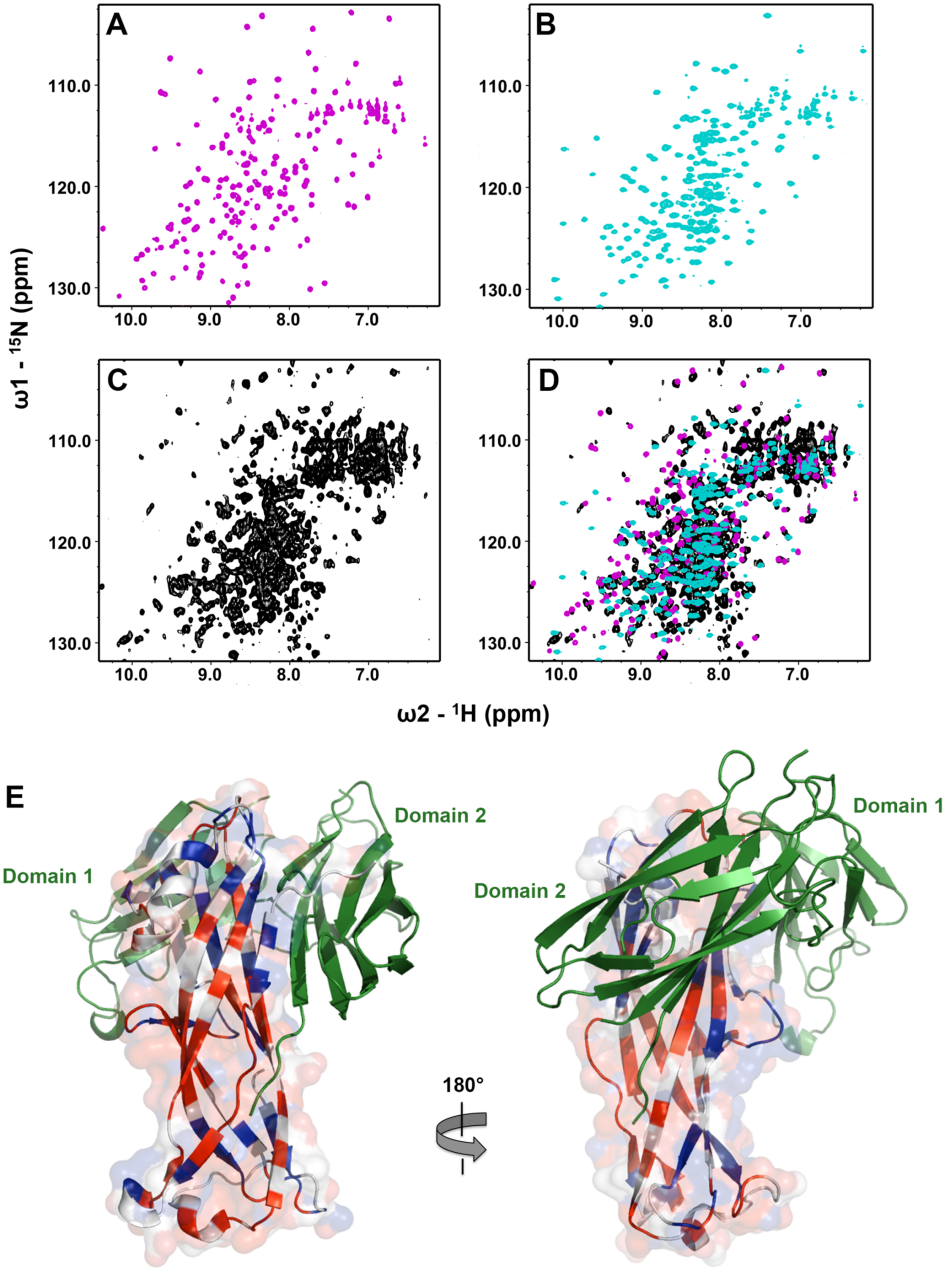
NMR analysis of the EcpB-EcpA^W11A^ complex. ^1^H-^15^N NMR HSQC spectra for (A) EcpAsc (magenta), (B) free EcpB (cyan), (C) EcpB-EcpA^W11A^ complex (black) and (D) all three spectra overlaid. (E) Model of the EcpB-EcpA^W11A^ complex based on the CsuC-CsuA/B structure. EcpB is coloured green, whilst EcpA is coloured based on chemical shift perturbations between ^1^H-^15^N NMR HSQC spectra of EcpAsc and the complex (grey: assignment could not be made; blue: Δ<0 line width; red: Δ>1 line width).

To confirm whether EcpB maintains EcpA in a partially folded conformation, as observed for the CsuC-CsuA/B complex, we used NMR to compare the solution states of self-complemented EcpA (EcpAsc) representing the final fibre-inserted conformation [6], free EcpB and the EcpB-EcpA^W11A^ complex (Fig 9). Inspection of 2D ^1^H-^15^N HSQC spectra for free EcpA and EcpB (Fig 9A and 9B) reveals excellent chemical shift dispersion with resonances observable for all non-proline amides, consistent with the fully folded domains observed in the crystal structures. However, the ^1^H-^15^N TROSY NMR spectrum of the EcpB-EcpA^W11A^ complex (Fig 9C) displays features characteristic of less ordered regions in the structure. This is highlighted by the high number of peaks at ~8.0 ppm when compared to the free components as well as significant variations in amide line-widths. To explore any conformational differences within the pre-assembly complex we transferred resonance assignments made on EcpAsc to the NMR spectrum of EcpB-EcpA^W11A^ [6]. Taking a conservative nearest neighbour approach we were able to assign only ~54% of the EcpA sequence within the complex (Fig 9D). We then measured chemical shift differences for EcpA resonances in free and bound spectra and mapped them onto a docked model based on the CsuC-CsuA/B structure (Fig 9E). Minor shifts of up to one line width difference were grouped together (blue) and those experiencing greater than one line width shift or were either broadened beyond detection or shifted beyond this were categorized as major shifts (red). Strikingly, major chemical shift perturbations are consistent with observations from the crystal structure of CsuC-CsuA/B, where they not only localise to regions in EcpA that interact directly with the chaperone, but more significantly many lie far from the EcpB interface and colocalise with same regions in CsuA/B, for which electron density was not observed. Furthermore, superimposing NMR spectra for the chaperone EcpB in its free form and the EcpB-EcpA^W11A^ complex (Fig 9D) reveals equally dramatic and widespread chemical shift differences, which is in stark contrast to results obtained from a similar NMR study on FimC-FimH from the classical Fim system [22]. In this study, chemical shift differences occurred at the direct interface with FimH and there was also an absence of any changes in D2 of FimC chaperone suggesting that the domain orientation is preserved. Our data on EcpB-EcpA^W11A^ suggest that the two domains in EcpB undergo a substantial reorientation upon formation of the complex. The subunit EcpA is trapped at an early folding intermediate in which a large portion of the structure remains conformationally heterogeneous and not ordered. Taken together our results suggest that alternative and archaic systems are closely related and define a new non-classical pathway, where their chaperones transport partially folded subunits to the usher. This is in contrast to the classical systems, where subunits are substantially folded, but maintained in an assembly competent conformation.

## Discussion

Our new structural and biochemical data on the non-classical CU pathway show that, although donor strand complementation governs specific chaperone-subunit and subunit-subunit interactions across all CU families, major differences in how the classical and non-classical pathways implement this mechanism exist.

It has been suggested that the chaperone-subunit association begins with the binding of the C-terminal end of the subunit to the inter-domain cleft of the chaperone [23]. The vital role of this step in the biogenesis of CU organelles explains why the carboxylate anchoring residues are among the most highly conserved residues in chaperones. However, the distinct differences between anchoring mechanism of archaic and classical chaperones suggest a large evolutionary distance between these systems. The striking feature of non-classical chaperones is the direct involvement of D2 in the anchoring mechanism. To fulfil this role, the domain must be precisely positioned in the subunit bound conformation of the chaperone. Interestingly, the relative orientation of the two chaperone domains in the archaic CsuC-CsuA/B and classical complexes (e.g. Caf1M-Caf1) is nearly identical (Figs 3A and 7B). In contrast, the domain orientation in subunit-free conformations can be varied significantly, as seen in the structure of EcpB (Fig 7B), which implies that a substantial rearrangement in the relative domain orientation occurs during the formation of the chaperone-subunit complex. Movement of the domains apart from one another would significantly decrease the affinity of the chaperone for the subunit in non-classical systems and hence may provide a mechanism for subunit release during the DSE assembly on the usher platform.

CU fibre assembly does not require energy from external sources. It has been shown that classical CU chaperones preserve a proportion of the folding free energy of subunits, which is later used to facilitate fibre formation [14,24]. Our data suggest that this free energy is, at least in part, stored in a new relative domain orientation adopted by the chaperone when in complex with the subunit. The relative motion of the two chaperone domains during subunit release could be a source of the physical force necessary for translocation of the fibre through the usher pore. The nature and the extent of the changes in domain orientation could be tuned for the size of the subunit secreted and the architecture of the final pilus. This would also explain why the adhesive tip of the ECP system (EcpD) has a dedicated chaperone, as the tip subunit is the largest of all the CU pathways at ~60 kDa.

Although the chaperone-bound subunits in classical systems are highly structured, they represent the high-energy intermediate and upon DSE the subunit undergoes a structural rearrangement [14] that releases this free energy [24]. Here, we have discovered that archaic and alternative chaperones, unlike their classical counterparts, maintain pilus subunits in a state that exhibits significant disorder. The structure of CsuA/B reveals a loosely packed hydrophobic core, resembling a folding intermediate such as a molten globule. As such, classical and non-classical chaperones appear to trap subunit-folding intermediates at very different stages. Most free energy of folding is released at the stage of hydrophobic core collapse, therefore the non-classical chaperones may preserve significantly more folding free energy of subunits.

In classical systems, the subunit assembly proceeds via an usher-coordinated, stepwise zip-in-zip-out mechanism. This involves the gradual replacement of donor strand G_1_ of the chaperone by free donor strand G_d_ of the incoming subunit [13,14], which is initiated by an insertion of a hydrophobic side chain at the C-terminal end of strand G_d_ into a vacant P5 pocket of the acceptor cleft [13,25,26]. Our study shows that subunits from non-classical systems are devoid of such a pre-folded DSE initiation site, as this region (P4-5) is disordered, which raises the important question of how the zip-in-zip-out process could be initiated in these systems?

In classical chaperone-subunit complexes the donor strand G_1_ from the chaperone is buried within the compactly folded subunit, however in the non-classical CsuC-CsuA/B complex, it is significantly more accessible to the solvent and only transiently covered by the poorly structured mini-strand A´. The attacking G_d_ strand could intercalate fully between strands B and G_1_, which could then be followed by the displacement of chaperone strand G_1_ by the subunit donor strand G_d_. In contrast to the classical zip-in-zip-out DSE, lateral replacement could start from any pocket of the acceptor cleft. Interestingly, such a mechanism would explain how the large side chain of Trp11 is introduced in the hydrophobic core of EcpA during DSE [6]. Classical systems possess a bulky hydrophobic residue at the C-terminal end of the donor strand, whereas the largest donor residue of EcpA, Trp11, is located centrally. The side chain of Trp11 is deeply inserted into pocket P2 of the cleft (S6A and S6B Fig) and it would seem more likely that this residue initiates DSE by attacking the disordered region of the subunit laterally (S8 Fig). In the following events, one half of the donor strand in EcpA (residues 1–13) may participate in zip-in-zip-out DSE and the other half (residues 14–17) could stabilise the folding of the remaining part of the subunit.

The partially unfolded nature of the chaperone-bound subunit in non-classical systems offers a highly flexible mechanism for assembly. DSE initiation could occur at lower pockets of the acceptor cleft than P5, which could be followed by a lateral replacement of the donor strand or by a combination of lateral replacement and the classical zip-in-zip-out mechanism. Adaptability of this system to accept larger side chains within the central region of the donor strand side chains could also increase the stability of the final assembled fibre. It is also conceivable that the classical mode of utilising folded subunits has evolved more recently and suggests that it may be a highly refined and more efficient assembly process. It should be noted, that although our study demonstrates a distinct “non-classical” mechanism for Csu and Ecp systems, other systems might show a more mixed type of assembly. For example, β-fimbriae, which are currently considered as classical systems, are in fact closer to the non-classical types described here. Classical subunit anchoring residues are absent in β-fimbriae chaperones, but analogous residues to the non-classical chaperones are present (S9 Fig).

Inhibiting the biogenesis of virulence pili at the level of periplasmic assembly is a highly promising strategy for the prevention of infections caused by antibiotic resistant Gram-negative pathogens. Here, we have shown that although the subunit C-terminal carboxylate-binding site is present in both classical and non-classical chaperones, its precise makeup is different between the two families. It may therefore require separate approaches to target this critical subunit-binding site with inhibitors of classical and non-classical CU pathways. At the same time, our discovery of the core-exposed conformation of the chaperone-bound subunit in non-classical systems suggests a novel inhibition strategy: potentially it should be possible to inhibit the DSE step of non-classical pathways by specific targeting of the accessible core. Another attractive target for inhibition is the usher-binding site on the chaperone [27]. Here, both classical and non-classical chaperones present a conserved hydrophobic surface for recruitment by the usher (S10 Fig), therefore, a broad range inhibitor could potentially be developed against this interaction.

## Methods

### Design of expression constructs

Expression plasmids were constructed using a procedure that we previously developed for the expression of fimbrial subunits [4]. Synthetic genes of CsuC fused to a 6His-tag (6H), CsuA/B and CsuE were ordered from GenScript. Each of the genes was delivered on plasmid pUC57. The DNA fragment coding for CsuC-6H was inserted into the expression pET101 vector (Invitrogen) using restriction enzyme sites *Eco*RI and *Sac*I to produce plasmid pET101-CsuC6H. 6H was removed by reverse PCR using primers CsuC-6Hdel-R and -F (S4 Table) to yield plasmid pET101-CsuC. The nucleotide sequence, encoding residues 29–37 of CsuA/B (residues 4–12 in the mature protein sequence), was replaced by a 6H-coding fragment with a reverse PCR using primers 6H-CsuAB-R and -F (S4 Table). The modified gene of CsuA/B (6HCsuA/B) was cut out with restriction enzymes *Nhe*I and *Sac*I and inserted into the same sites in pET101-CsuC to create the CsuC and 6HCsuA/B co-expression plasmid pET101-CsuC-6HCsuA/B. To produce plasmids pET101-Csu6H-CsuA/B and pET101-Csu6H-CsuE, co-expressing CsuC6H with wild type subunits CsuA/B and CsuE, respectively, the CsuA/B or CsuE genes were cloned into plasmid pET101-CsuC6H using restriction sites for *Nhe*I and *Sac*I. Full-length *ecpB* including the N-terminal periplasmic signal sequence and incorporating a C-terminal His-tag was cloned into the pET28b vector (pET28*ecpB*) using In-Fusion (Clontech). Full-length *ecpA* including the N-terminal periplasmic signal sequence was cloned into the pBAD vector using In-Fusion (Clontech). Trp11 was then mutated to an alanine to prevent auto-aggregation using reverse PCR (pBAD*ecpA* ^*W11A*^). All oligonucleotides are listed in S4 Table.

### Mutagenesis

Mutagenesis of CsuC and CsuA/B genes in plasmids pET101-CsuC-6HCsuA/B and pET101-CsuC6H-CsuA/B, respectively, and EcpB in plasmid pET28, were performed by reverse PCR using primers listed in S4 Table.

### Protein expression and purification

CsuC-CsuA/B expression and purification is described in [28]. EcpB expression and purification is described in [29]. Wild type and mutant EcpB-EcpA^W11A^ complexes were expressed by co-transformation of *E*. *coli* BL21 (DE3) with pET28*ecpB* and pBAD*ecpA* ^*W11A*^, followed by growth in LB media at 37°C, induction at OD_600nm_ 0.6 with 0.5 mM IPTG and 0.05% L-arabinose, and incubation overnight at 18°C. For ^15^N-labelled samples, expression was undertaken in M9 media supplemented with ^15^NH_4_Cl. Cells were harvested by centrifugation and samples purified by Ni^2+^-affinity chromatography. NMR samples were further purified with an S200 gel filtration column (GE healthcare).

### Crystal structure determination

Determination of the crystal structure of CsuC:CsuA/B is described in [28]. Model building and refinements were performed by PHENIX refinement module. Manual corrections were done with molecular modelling program COOT (Emsley P., et al., 2010). Crystals of EcpB were obtained as described in (Garnett *et al*., 2015). Derivative crystals were obtained by soaking native crystals for 30s in 0.5 M NaI, 15% (*v*/*v*) glycerol, 15% (*w*/*v*) PEG 5000 MME prior to freezing. I-SAD data were collected and initial phases were calculated by SIRAS using SHELXD and SHARP. Automated model building was carried out with ArpWarp, refinement with Refmac and manual model building in COOT.

### NMR spectroscopy

^1^H -^15^N TROSY NMR spectra were collected on ^15^N-labelled samples (250 μM CsuC-CsuCA/B complex, 150 μM EcpB-EcpA^W11A^ complex) in 20 mM HEPES pH 7.0, 100 mM NaCl, 10% D_2_O at 298K on a Bruker Avance II 800 spectrometer equipped with TCI cryoprobe. A ^1^H-^15^N HSQC NMR spectrum of free EcpB (250 μM) in the same buffer was collected at 298K on a Bruker Avance III 600 spectrometer equipped with TCI cryoprobe. Data were analysed with in-house script using NMRview [30].

### Accession numbers

The coordinates and structure factors have been deposited in the Protein Data Bank under accession codes 5D6H and 5DFK for the CsuC-CsuA/B complex and EcpB chaperone, respectively.

## Supporting Information

S1 Fig Alignment of sequences of archaic and alternative pathway chaperones. Periodic structure (rectangle, α-helix; arrow, β-strand) is shown above the amino acid sequences of CsuC and CfaA. Invariant proline is shown in red and highly conserved positions are indicated by background shading in cyan. Donor residues and residues anchoring subunit carboxylate are indicated by background shading in yellow and green, respectively. Residues predicted to mediate usher binding are shown by shading in grey. Residues that form ionic or hydrogen bonds with the super-conserved arginine, anchoring C-terminal carboxylate of subunits, are shown by shading in blue (S11 Fig). CLUSTALW alignment of sequences was modified based on superposition of structures of CsuC (this study), EcpB (this study) and CfaA [20].
(PDF)

S2 Fig Crystal structure of CsuC:A/B (stereo diagram). CsuC and CsuA/B are painted in cyan and magenta, respectively. Donor residues (Val110, Phe112, Met114, Tyr116) in strand G_1_ and additional subunit binding residues in strand A (Phe3, Leu4, Ile5, Trp6, Pro7, Ile8, Tyr9, Pro10) and C-terminal carboxylate of CsuA/B anchoring residues (Arg89, Tyr196, and Arg174) are shown as balls-and-sticks. N and C termini and β-strands are labelled. The asterisk in the *N´ label indicates that the N-terminal donor sequence of CsuA/B has been replaced by a His-tag.
(PDF)

S3 Fig Gel filtration chromatography (S200 column GE healthcare) of periplasmic purified EcpB-EcpA complex. EcpB-EcpA elutes as a globular protein with molecular weight of about 50 kDa, which corresponds to a 1:1 stoichiometry for the complex. The SDS-PAGE analysis of selected elution fractions shows bands of EcpA and EcpB. The band of EcpA has slightly lower intensity than that of EcpB. This is presumably because EcpA is 26% smaller than EcpB.
(PDF)

S4 Fig Phylogenetic analysis of periplasmic chaperones from the alternative chaperone-usher pathway (maximum likelihood tree). The scale bar represents 0.1 substitutions per site. Magenta, EcpB-like chaperones; green, CfaA-like chaperones.
(PDF)

S5 Fig Yields of EcpAW11A and wild type EcpB or EcpB mutants. Levels based on SDS-PAGE analysis of purified of EcpA and EcpB have been scaled relative to EcpB WT.
(PDF)

S6 Fig Donor strand exchange in ECP system. (**A**) Superposition of CsuA/B (magenta) and donor strand complemented (dsc) EcpA (cyan except for donor strand G_d_, which is shown in green). Donor strand residues in dscEcpA are shown as balls-and-sticks. N and C termini and β-strands are labelled. (**B**) Comparison of donor strand complementation of EcpA in ECP pili with that proposed by the EcpB chaperone. EcpA without the donor strand is shown as molecular surface. Seven hydrophobic pockets in the donor-strand binding cleft are labelled from P-1 to P5. Donor strand G_d_ of EcpA is shown on the right. Model of the donor strand segment in strand G_1_ of EcpB is shown on the left. Residues that are involved in donor-strand exchange are labelled. The EcpB donor residues-pockets assignment was determined based on superpositions of EcpA and CsuA/B (A) and EcpB and CsuC (Fig 5B and 5C). Residues 111–113 are disordered in the crystal structure of subunit-free EcpB. To generate the figure, this segment was modelled based on the corresponding region in CsuC form the crystal structure of the CsuC-CsuA/B complex.
(PDF)

S7 Fig The super-conserved proline in periplasmic chaperones forms a sharp kink in the polypeptide chain, switching strand D_1_ between the β-sheets of domain 1. Classical Caf1M (yellow), archaic CsuC (cyan), and alternative EcpB (purple) and CfaA (green) chaperones were superimposed as in Fig 5B. The cartoon diagram shows the D_1_´-D_1_´´ fragment of the superposition. The proline is shown as ball-and-sticks.
(PDF)

S8 Fig Model for initiation of the lateral replacement donor strand exchange (DSE). The topology diagram depicts a chaperone-bound subunit from a non-classical system, as revealed in the crystal structure of the CsuC-CsuA/B complex. Unstructured segments of the subunit are not shown, except for the N-terminal donor strand extension indicated in a dashed line. Donor strand G_d_ of the attacking subunit is shown as thick black line with balls illustrating hydrophobic donor residues. Strand G_d_ intercalates between strands B and G_1_, occupying the open cleft in the loosely packed subunit. At the next step, a lager donor strand residue in strand G_d_ (such as e.g. Trp11 in EcpA) moves laterally and replaces a donor strand residue in strand G_1_, starting DSE. After the DSE completion, strand G_d_ occupies the acceptor cleft instead of strand G_1_ and subunit folds in a compact structure.
(PDF)

S9 Fig Alignment of sequences of chaperones involved in β-fimbriae assembly and CsuC. Periodic structure (rectangle, α-helix; arrow, β-strand) is shown above the amino acid sequences of CsuC. Stars indicate three residues in CsuC that anchor subunit carboxylate. The same or a similar type of residue (shown by background shading in green) occupies these positions in β-fimbriae chaperones, suggesting that they use the non-classical mechanism to anchor pilus subunits. Donor residues in CsuC and predicted hydrophobic donor residues in β-fimbriae chaperones are shown with background shading in yellow. Note that in β-fimbriae chaperones, a polar residue (Gln) occupies position P0. This indicates that β-fimbriae chaperones have no the C-terminal shift of the donor strand motif. Invariant residues that are not implicated in subunit binding are shown by background shading in cyan. The alignment was produced by CLUSTALW.
(PDF)

S10 Fig Prediction of usher-binding surface in CsuC and EcpB. N-terminal domains of CsuC and Caf1M (A) and EcpB and Caf1M (B) were superimposed by distance minimization between Cα atoms of corresponding residues. Usher-binding residues in Caf1M [31] and hydrophobic surface residues in CsuC and EcpB are shown as balls-and-sticks. CsuC, EcpB, and Caf1M are coloured in cyan, magenta, and bronze, respectively. CsuC has large hydrophobic residues in all positions mediating the binding to the usher in classical chaperones. The hydrophobic nature of these positions in archaic chaperones (S2 Fig) suggests that archaic and classical systems employ similar mechanisms for the selective binding of pre-assembly complexes to the usher. As CsuC and Caf1M, EcpB possess several hydrophobic residues on the surface of the β-sheet G_1_F_1_C_1_D_1_. Leu43 in EcpB occupies a hydrophobic position that is conserved in both classical (Met32 in CsuC) and non-classical (Leu43 in Caf1M) chaperones and is implicated in the usher binding in classical chaperones. The hydrophobic position corresponding to Leu67 in Caf1M (Val61 in CsuC) is not present in alternative chaperones. Instead, they contain a conserved hydrophobic position located two residues downstream, which, in EcpB, is occupied by surface exposed Leu79. Hence, alternative chaperones also possess a hydrophobic patch, which they likely use to bind to the usher.
(PDF)

S11 Fig Super-conserved subunit C-terminus-binding arginine in non-classical chaperones is stabilised by an ionic or hydrogen bond to a neighbouring residue. Fragments of structures of CsuC, EcpB, and CfaA demonstrate interaction of the subunit C-terminus anchoring arginine with neighbouring residues. CsuC: hydrogen bond between Arg89 and conserved Ser117 in archaic chaperones; EcpB: ionic bond between Arg89 and Glu36; CfaA: ionic bond between Arg90 and conserved Glu48 in CfaA-like chaperones. Residues are shown as balls-and-sticks.
(PDF)

S1 Table Diffraction data and refinement statistics. (PDF)

S2 Table Structural comparison of periplasmic chaperones. (PDF)

S3 Table Fraction of structured sequence in pre-assembly complexes. (PDF)

S4 Table Oligonucleotides. (PDF)
