# Supplementary material for: Structural Insight into Archaic and Alternative Chaperone-Usher Pathways Reveals a Novel Mechanism of Pilus Biogenesis
Source: PLoS Pathog. 2015 Nov 20;11(11):e1005269. doi: 10.1371/journal.ppat.1005269 (PMC4654587; doi:10.1371/journal.ppat.1005269)
Supplement: S1 Fig — Periodic structure (rectangle, α-helix; arrow, β-strand) is shown above the amino acid sequences of CsuC and CfaA. Invariant proline is shown in red and highly conserved positions are indicated by background shading in cyan. Donor residues and residues anchoring subunit carboxylate are indicated by background shading in yellow and green, respectively. Residues predicted to mediate usher binding are shown by shading in grey. Residues that form ionic or hydrogen bonds with the super-conserved arginine, anchoring C-terminal carboxylate of subunits, are shown by shading in blue (S11 Fig). CLUSTALW alignment of sequences was modified based on superposition of structures of CsuC (this study), EcpB (this study) and CfaA [20]. (PDF) [file ppat.1005269.s001.pdf]

S1 Fig.

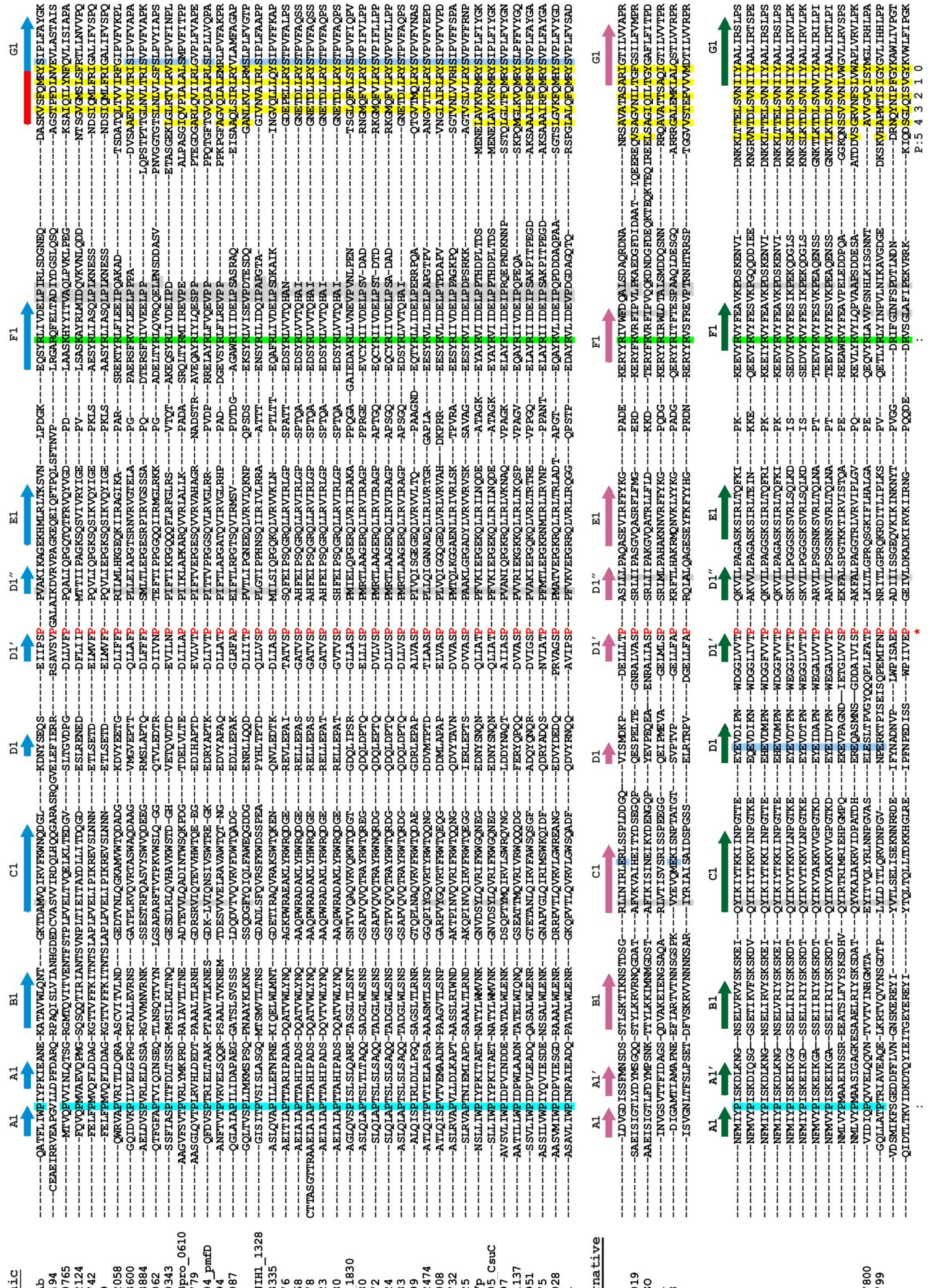
Archaic
CsuC Ab
HNE_2494
Saro_0765
PSHAa2124
VV1_2742
VV1519
Pcar_2058
Adeh_3600
MXAN_3884
DR_B0062
Bxe_B0343
PmfD_Bpro_0610
Mlg_0779
MCA0304_pmfD
RSc2694
HNE_0087
CsuC
GbCGDNIH1_1328
ACIAD3335
XCC1376
XOO1868
XOO1978
XAC1423
XCV1480
Pcryo_1830
XCC1380
XOO1872
XAC1424
XCV1483
Rsp1499
Bamb_2474
BPSL1008
Atu_4732
mll7725
CsuC_Vp
VPA1505_CsuC
YPO1697
EFER_1137
PFL_3951
ECA3075
BPSL2028
PA4651
Alternative
EcpB
AHA_1019
CooB_SO
AHA_61
AHA_58
EcpE
CfaA
CotB
CsaA
CsuB
CooB
CosB
CsbB
CsdB
TcfA
CblB
YPO_37800
YPO_3799
CsfB
CsfE
A1
A1′
B1
C1
D1
D1′
D1″
E1
F1
G1
P:5 4 3 2 1 0

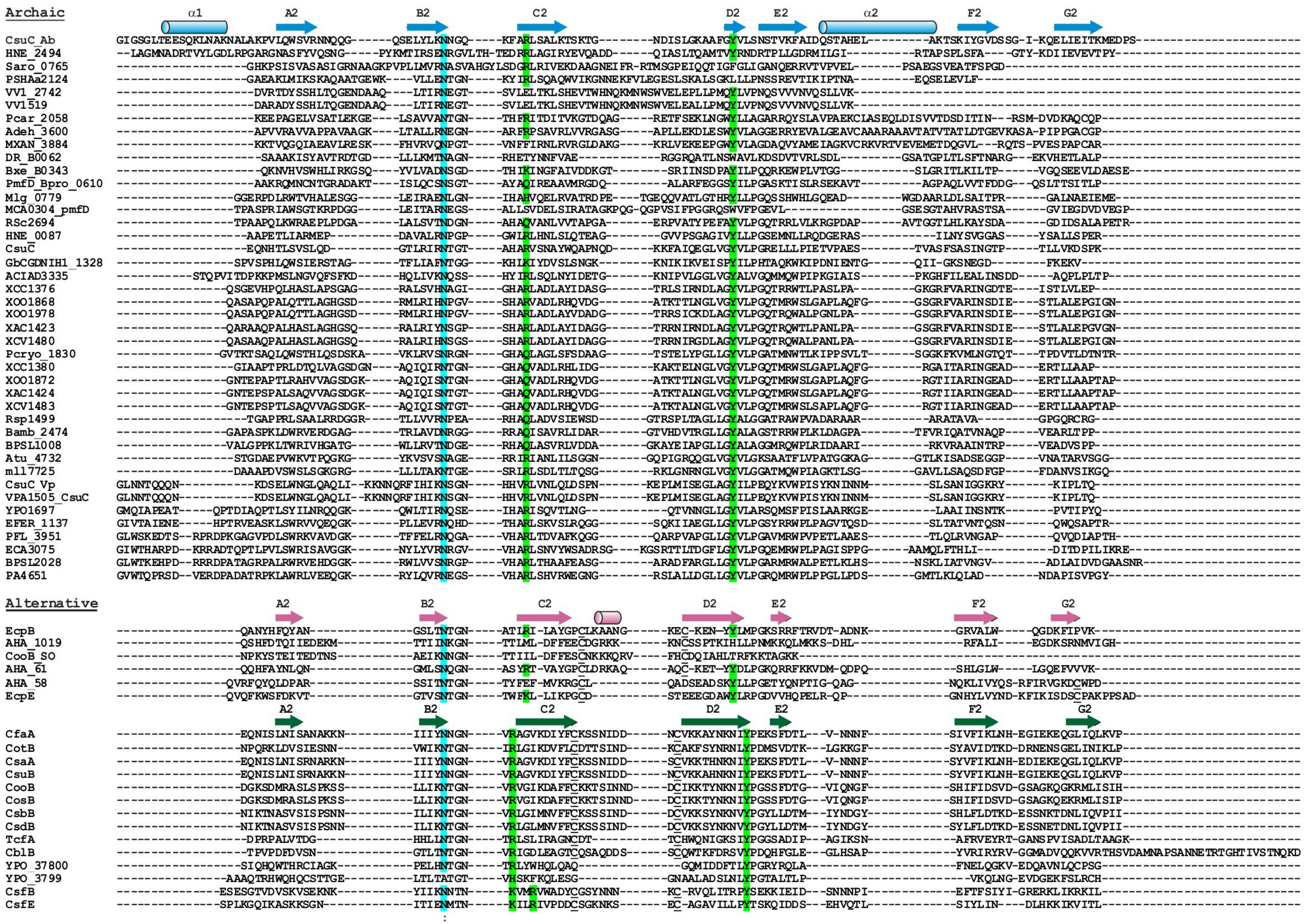
Archaic
α1
A2
B2
C2
D2
E2
α2
F2
G2
CsuC_Ab
HNE_2494
Saro_0765
PSHAa2124
VV1_2742
VV1519
Pcar_2058
Adeh_3600
MXAN_3884
DR_B0062
Bxe_B0343
PmfD_Bpro_0610
Mlg_0779
MCA0304_pmfD
RSc2694
HNE_0087
CsuC
GbCGDNIH1_1328
ACIAD3335
XCC1376
XOO1868
XOO1978
XAC1423
XCV1480
Pcryo_1830
XCC1380
XOO1872
XAC1424
XCV1483
Rsp1499
Bamb_2474
BPSL1008
Atu_4732
mll7725
CsuC_Vp
VPA1505_CsuC
YPO1697
EFER_1137
PFL_3951
ECA3075
BPSL2028
PA4651
Alternative
EcpB
AHA_1019
CooB_SO
AHA_61
AHA_58
EcpE
CfaA
CotB
CsaA
CsuB
CooB
CosB
CsbB
CsdB
TcfA
CblB
YPO_37800
YPO_3799
CsfB
CsfE

Alignment of sequences of archaic and alternative pathway chaperones. Periodic structure (rectangle, α-helix; arrow, β-strand) is shown above the amino acid sequences of CsuC and CfaA. Invariant proline is shown in red and highly conserved positions are indicated by background shading in cyan. Donor residues and residues anchoring subunit carboxylate are indicated background shading in yellow and green, respectively. Residues predicted to mediate usher binding are shown by shading in grey. Residues that form ionic or hydrogen bonds with the super-conserved arginine, anchoring C-terminal carboxylate of subunits, are shown by shading in blue (S11 Fig.). CLUSTALW alignment of sequences was modified based on superposition of structures of CsuC (this study), EcpB (this study) and CfaA (Bao et al., 2014).
