# Supplementary material for: Structural Insight into Archaic and Alternative Chaperone-Usher Pathways Reveals a Novel Mechanism of Pilus Biogenesis
Source: PLoS Pathog. 2015 Nov 20;11(11):e1005269. doi: 10.1371/journal.ppat.1005269 (PMC4654587; doi:10.1371/journal.ppat.1005269)
Supplement: S2 Fig — CsuC and CsuA/B are painted in cyan and magenta, respectively. Donor residues (Val110, Phe112, Met114, Tyr116) in strand G1 and additional subunit binding residues in strand A (Phe3, Leu4, Ile5, Trp6, Pro7, Ile8, Tyr9, Pro10) and C-terminal carboxylate of CsuA/B anchoring residues (Arg89, Tyr196, and Arg174) are shown as balls-and-sticks. N and C termini and β-strands are labelled. The asterisk in the *N´ label indicates that the N-terminal donor sequence of CsuA/B has been replaced by a His-tag. (PDF) [file ppat.1005269.s002.pdf]

S2 Fig.

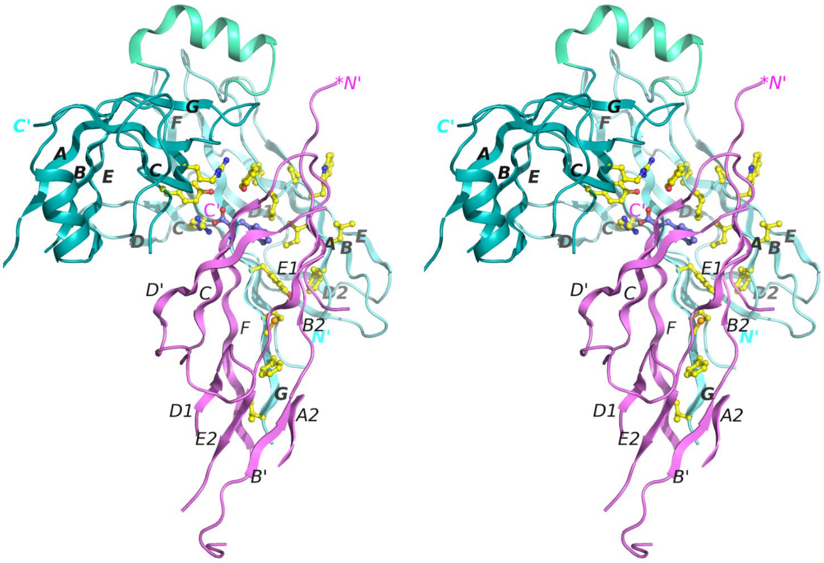

Crystal structure of CsuC:A/B (stereo diagram). CsuC and CsuA/B are painted in cyan and magenta, respectively. Donor residues (Val110, Phe112, Met114, Tyr116) in strand G_1 and additional subunit binding residues in strand A (Phe3, Leu4, Ile5, Trp6, Pro7, Ile8, Tyr9, Pro10) and C-terminal carboxylate of CsuA/B anchoring residues (Arg89, Tyr196, and Arg174) are shown as balls-and-sticks. N and C termini and β-strands are labeled. The asterisk in the *N´ label indicates that the N-terminal donor sequence of CsuA/B has been replaced by a His-tag.
