# Supplementary material for: Structural Insight into Archaic and Alternative Chaperone-Usher Pathways Reveals a Novel Mechanism of Pilus Biogenesis
Source: PLoS Pathog. 2015 Nov 20;11(11):e1005269. doi: 10.1371/journal.ppat.1005269 (PMC4654587; doi:10.1371/journal.ppat.1005269)
Supplement: S3 Fig — EcpB-EcpA elutes as a globular protein with molecular weight of about 50 kDa, which corresponds to a 1:1 stoichiometry for the complex. The SDS-PAGE analysis of selected elution fractions shows bands of EcpA and EcpB. The band of EcpA has slightly lower intensity than that of EcpB. This is presumably because EcpA is 26% smaller than EcpB. (PDF) [file ppat.1005269.s003.pdf]

S3 Fig.

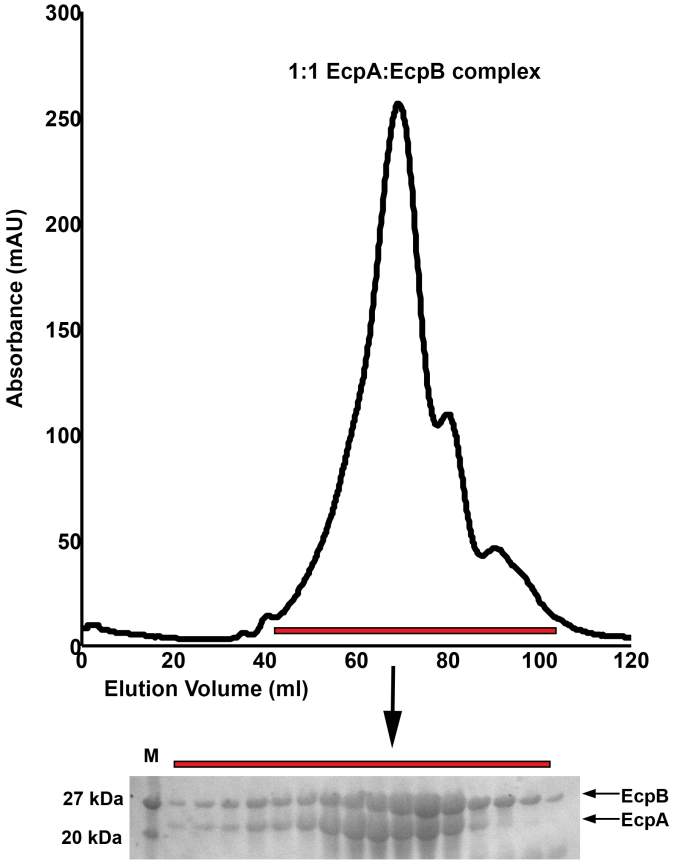

Gel filtration chromatography (S200 column GE healthcare) of periplasmic purified EcpB-EcpA complex. EcpB-EcpA elutes as a globular protein with molecular weight of about 50 kDa, which corresponds to a 1:1 stoichiometry for the complex. The SDS-PAGE analysis of selected elution fractions shows bands of EcpA and EcpB. The band of EcpA has slightly lower intensity than that of EcpB. This is presumably because EcpA is 26% smaller than EcpB.
