# Supplementary material for: Structural Insight into Archaic and Alternative Chaperone-Usher Pathways Reveals a Novel Mechanism of Pilus Biogenesis
Source: PLoS Pathog. 2015 Nov 20;11(11):e1005269. doi: 10.1371/journal.ppat.1005269 (PMC4654587; doi:10.1371/journal.ppat.1005269)
Supplement: S4 Fig — The scale bar represents 0.1 substitutions per site. Magenta, EcpB-like chaperones; green, CfaA-like chaperones. (PDF) [file ppat.1005269.s004.pdf]

**S4 Fig.**

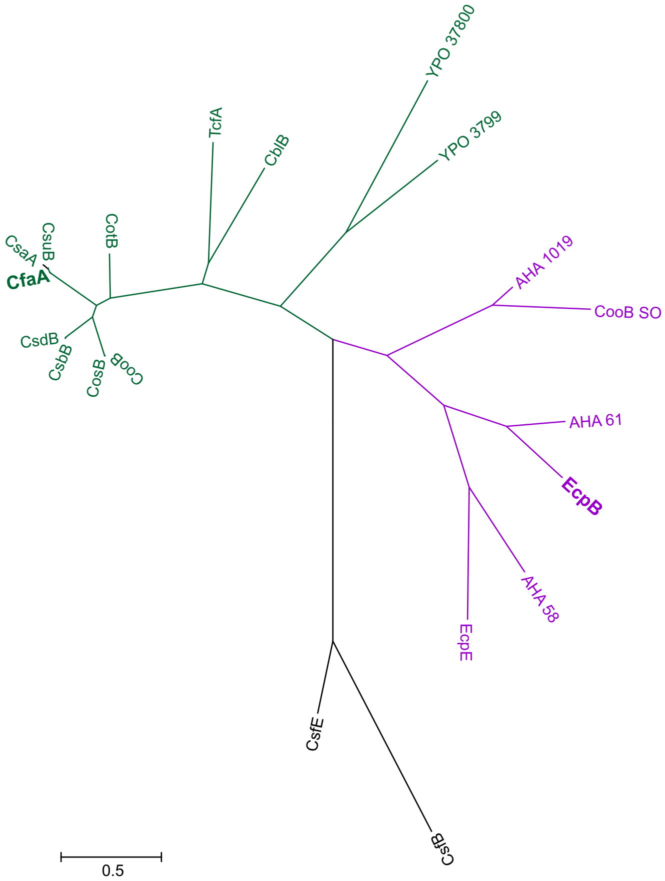


**Phylogenetic analysis of periplasmic chaperones from the alternative chaperone-usher pathway** (maximum likelihood tree). The scale bar represents 0.1 substitutions per site. Magenta, EcpB-like chaperones; green, CfaA-like chaperones.
