# Supplementary material for: Structural Insight into Archaic and Alternative Chaperone-Usher Pathways Reveals a Novel Mechanism of Pilus Biogenesis
Source: PLoS Pathog. 2015 Nov 20;11(11):e1005269. doi: 10.1371/journal.ppat.1005269 (PMC4654587; doi:10.1371/journal.ppat.1005269)
Supplement: S5 Fig — Levels based on SDS-PAGE analysis of purified of EcpA and EcpB have been scaled relative to EcpB WT. (PDF) [file ppat.1005269.s005.pdf]

S5 Fig.

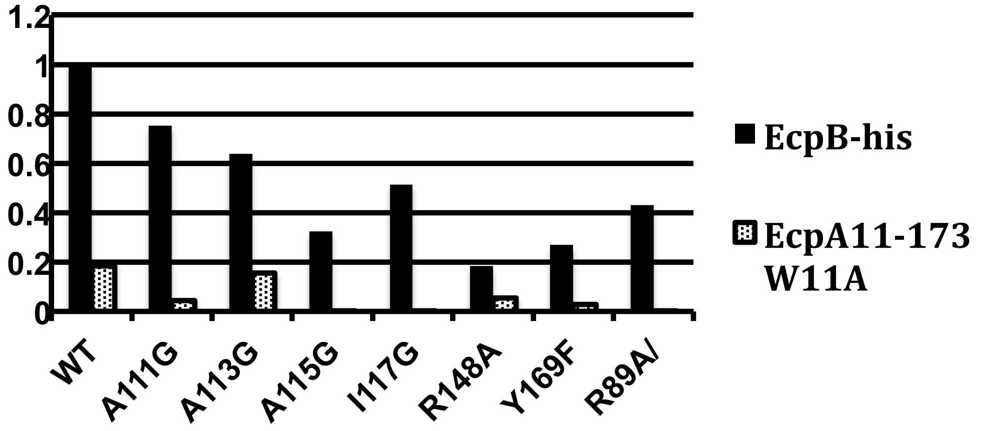

Yields of EcpAW11A and wild type EcpB or EcpB mutants. Levels based on SDS-PAGE analysis of purified of EcpA and EcpB have been scaled relative to EcpB WT.
