# Supplementary material for: Structural Insight into Archaic and Alternative Chaperone-Usher Pathways Reveals a Novel Mechanism of Pilus Biogenesis
Source: PLoS Pathog. 2015 Nov 20;11(11):e1005269. doi: 10.1371/journal.ppat.1005269 (PMC4654587; doi:10.1371/journal.ppat.1005269)
Supplement: S6 Fig — (A) Superposition of CsuA/B (magenta) and donor strand complemented (dsc) EcpA (cyan except for donor strand Gd, which is shown in green). Donor strand residues in dscEcpA are shown as balls-and-sticks. N and C termini and β-strands are labelled. (B) Comparison of donor strand complementation of EcpA in ECP pili with that proposed by the EcpB chaperone. EcpA without the donor strand is shown as molecular surface. Seven hydrophobic pockets in the donor-strand binding cleft are labelled from P-1 to P5. Donor strand Gd of EcpA is shown on the right. Model of the donor strand segment in strand G1 of EcpB is shown on the left. Residues that are involved in donor-strand exchange are labelled. The EcpB donor residues-pockets assignment was determined based on superpositions of EcpA and CsuA/B (A) and EcpB and CsuC (Fig 5B and 5C). Residues 111–113 are disordered in the crystal structure of subunit-free EcpB. To generate the figure, this segment was modelled based on the corresponding region in CsuC form the crystal structure of the CsuC-CsuA/B complex. (PDF) [file ppat.1005269.s006.pdf]

S6 Fig.

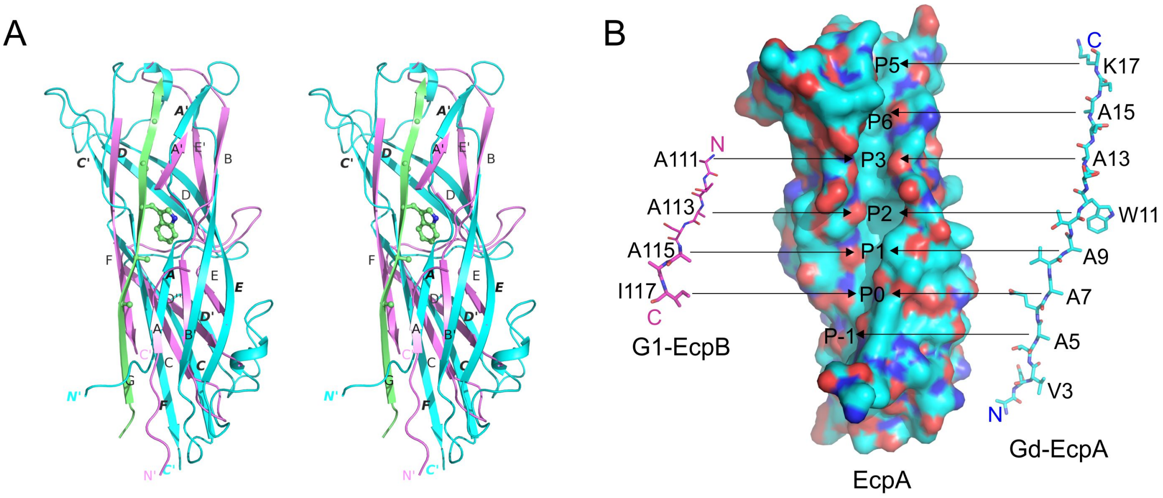

Donor strand exchange in ECP system. (**A**) Superposition of CsuA/B (magenta) and donor strand complemented (dsc) EcpA (cyan except for donor strand G_d, which is shown in green). Donor strand residues in dscEcpA are shown as balls-and-sticks. N and C termini and β-strands are labeled. (**B**) Comparison of donor strand complementation of EcpA in ECP pili with that proposed by the EcpB chaperone. EcpA without the donor strand is shown as molecular surface. Seven hydrophobic pockets in the donor-strand binding cleft are labeled from P-1 to P5. Donor strand G_d of EcpA is shown on the right. Model of the donor strand segment in strand G_1 of EcpB is shown on the left. Residues that are involved in donor-strand exchange are labeled. The EcpB donor residues-pockets assignment was determined based on superpositions of EcpA and CsuA/B (A) and EcpB and CsuC (Figure 5B and C). Residues 111-113 are disordered in the crystal structure of subunit-free EcpB. To generate the figure, this segment was modeled based on the corresponding region in CsuC form the crystal structure of the CsuC-CsuA/B complex.
