# Supplementary material for: Structural Insight into Archaic and Alternative Chaperone-Usher Pathways Reveals a Novel Mechanism of Pilus Biogenesis
Source: PLoS Pathog. 2015 Nov 20;11(11):e1005269. doi: 10.1371/journal.ppat.1005269 (PMC4654587; doi:10.1371/journal.ppat.1005269)
Supplement: S7 Fig — Classical Caf1M (yellow), archaic CsuC (cyan), and alternative EcpB (purple) and CfaA (green) chaperones were superimposed as in Fig 5B. The cartoon diagram shows the D1´-D1´´ fragment of the superposition. The proline is shown as ball-and-sticks. (PDF) [file ppat.1005269.s007.pdf]

**S7 Fig.**

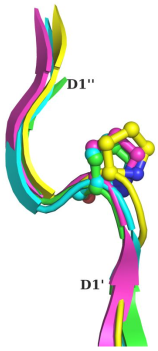


**The super-conserved proline in periplasmic chaperones forms a sharp kink in the polypeptide chain, switching strand $D_1$ between the β-sheets of domain 1**. Classical Caf1M (yellow), archaic CsuC (cyan), and alternative EcpB (purple) and CfaA (green) chaperones were superimposed as in Figure 5B. The cartoon diagram shows the $D_1'$-$D_1''$ fragment of the superposition. The proline is shown as ball-and-sticks.
