# Supplementary material for: Structural Insight into Archaic and Alternative Chaperone-Usher Pathways Reveals a Novel Mechanism of Pilus Biogenesis
Source: PLoS Pathog. 2015 Nov 20;11(11):e1005269. doi: 10.1371/journal.ppat.1005269 (PMC4654587; doi:10.1371/journal.ppat.1005269)
Supplement: S8 Fig — The topology diagram depicts a chaperone-bound subunit from a non-classical system, as revealed in the crystal structure of the CsuC-CsuA/B complex. Unstructured segments of the subunit are not shown, except for the N-terminal donor strand extension indicated in a dashed line. Donor strand Gd of the attacking subunit is shown as thick black line with balls illustrating hydrophobic donor residues. Strand Gd intercalates between strands B and G1, occupying the open cleft in the loosely packed subunit. At the next step, a lager donor strand residue in strand Gd (such as e.g. Trp11 in EcpA) moves laterally and replaces a donor strand residue in strand G1, starting DSE. After the DSE completion, strand Gd occupies the acceptor cleft instead of strand G1 and subunit folds in a compact structure. (PDF) [file ppat.1005269.s008.pdf]

S8 Fig.

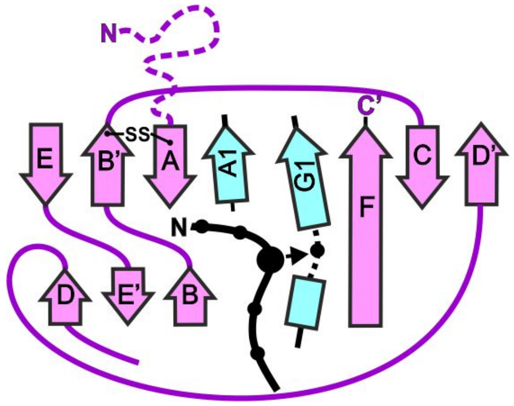

Model for initiation of the lateral replacement donor strand exchange (DSE). The topology diagram depicts a chaperone-bound subunit from a non-classical system, as revealed in the crystal structure of the CsuC-CsuA/B complex. Unstructured segments of the subunit are not shown, except for the N-terminal donor strand extension indicated in a dashed line. Donor strand G_d of the attacking subunit is shown as thick black line with balls illustrating hydrophobic donor residues. Strand G_d intercalates between strands B and G_1, occupying the open cleft in the loosely packed subunit. At the next step, a lager donor strand residue in strand G_d (such as e.g. Trp11 in EcpA) moves laterally and replaces a donor strand residue in strand G_1, starting DSE. After the DSE completion, strand G_d occupies the acceptor cleft instead of strand G_1 and subunit folds in a compact structure.
