# Supplementary material for: Structural Insight into Archaic and Alternative Chaperone-Usher Pathways Reveals a Novel Mechanism of Pilus Biogenesis
Source: PLoS Pathog. 2015 Nov 20;11(11):e1005269. doi: 10.1371/journal.ppat.1005269 (PMC4654587; doi:10.1371/journal.ppat.1005269)
Supplement: S9 Fig — Periodic structure (rectangle, α-helix; arrow, β-strand) is shown above the amino acid sequences of CsuC. Stars indicate three residues in CsuC that anchor subunit carboxylate. The same or a similar type of residue (shown by background shading in green) occupies these positions in β-fimbriae chaperones, suggesting that they use the non-classical mechanism to anchor pilus subunits. Donor residues in CsuC and predicted hydrophobic donor residues in β-fimbriae chaperones are shown with background shading in yellow. Note that in β-fimbriae chaperones, a polar residue (Gln) occupies position P0. This indicates that β-fimbriae chaperones have no the C-terminal shift of the donor strand motif. Invariant residues that are not implicated in subunit binding are shown by background shading in cyan. The alignment was produced by CLUSTALW. (PDF) [file ppat.1005269.s009.pdf]

S9 Fig.

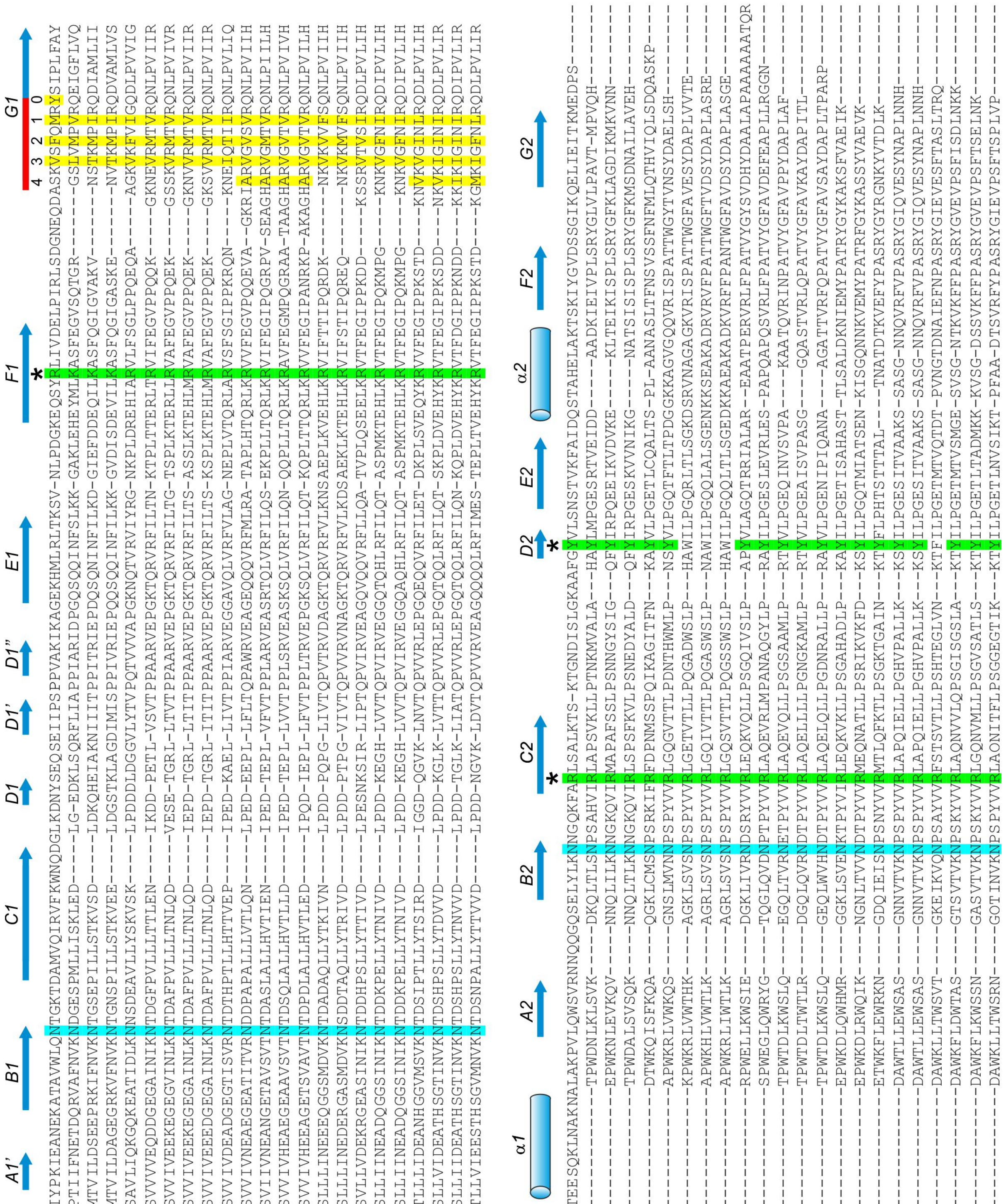

A1
A1'
B1
C1
D1
D1'
D1''
E1
F1
G1
4 3 2 1 0
α1
A2
B2
C2
D2
E2
α2
F2
G2
CsuC_Ab
BURPS1106A_RS18320
BB2000_RS15060
S70_RS03625
H045_RS09065
M495_RS19810
ETA_RS09375
EAM_RS08805
EPC_RS08915
Bamb_1978
PPUTGB1_RS05795
PPUTUW4_RS22555
PFLA506_RS04935
PFLU_1011
CKO_03114
ECL_05085
BWG_RS16115
ESA_RS16175
ES15_RS17375
ECWSU1_RS02995
ECL_04606
ECENHK_RS20050
SU5_RS03365

Alignment of sequences of chaperones involved in β-fimbriae assembly and CsuC. Periodic structure (rectangle, α-helix; arrow, β-strand) is shown above the amino acid sequences of CsuC. Stars indicate three residues in CsuC that anchor subunit carboxylate. The same or a similar type of residue (shown by background shading in green) occupies these positions in β-fimbriae chaperones, suggesting that they use the non-classical mechanism to anchor pilus subunits. Donor residues in CsuC and predicted hydrophobic donor residues in β-fimbriae chaperones are shown with background shading in yellow. Note that in β-fimbriae chaperones, a polar residue (Gln) occupies position P0. This indicates that β-fimbriae chaperones have no the C-terminal shift of the donor strand motif. Invariant residues that are not implicated in subunit binding are shown by background shading in cyan. The alignment was produced by CLUSTALW.
