# Supplementary material for: Structural Insight into Archaic and Alternative Chaperone-Usher Pathways Reveals a Novel Mechanism of Pilus Biogenesis
Source: PLoS Pathog. 2015 Nov 20;11(11):e1005269. doi: 10.1371/journal.ppat.1005269 (PMC4654587; doi:10.1371/journal.ppat.1005269)
Supplement: S10 Fig — N-terminal domains of CsuC and Caf1M (A) and EcpB and Caf1M (B) were superimposed by distance minimization between Cα atoms of corresponding residues. Usher-binding residues in Caf1M [31] and hydrophobic surface residues in CsuC and EcpB are shown as balls-and-sticks. CsuC, EcpB, and Caf1M are coloured in cyan, magenta, and bronze, respectively. CsuC has large hydrophobic residues in all positions mediating the binding to the usher in classical chaperones. The hydrophobic nature of these positions in archaic chaperones (S2 Fig) suggests that archaic and classical systems employ similar mechanisms for the selective binding of pre-assembly complexes to the usher. As CsuC and Caf1M, EcpB possess several hydrophobic residues on the surface of the β-sheet G1F1C1D1. Leu43 in EcpB occupies a hydrophobic position that is conserved in both classical (Met32 in CsuC) and non-classical (Leu43 in Caf1M) chaperones and is implicated in the usher binding in classical chaperones. The hydrophobic position corresponding to Leu67 in Caf1M (Val61 in CsuC) is not present in alternative chaperones. Instead, they contain a conserved hydrophobic position located two residues downstream, which, in EcpB, is occupied by surface exposed Leu79. Hence, alternative chaperones also possess a hydrophobic patch, which they likely use to bind to the usher. (PDF) [file ppat.1005269.s010.pdf]

S10 Fig.

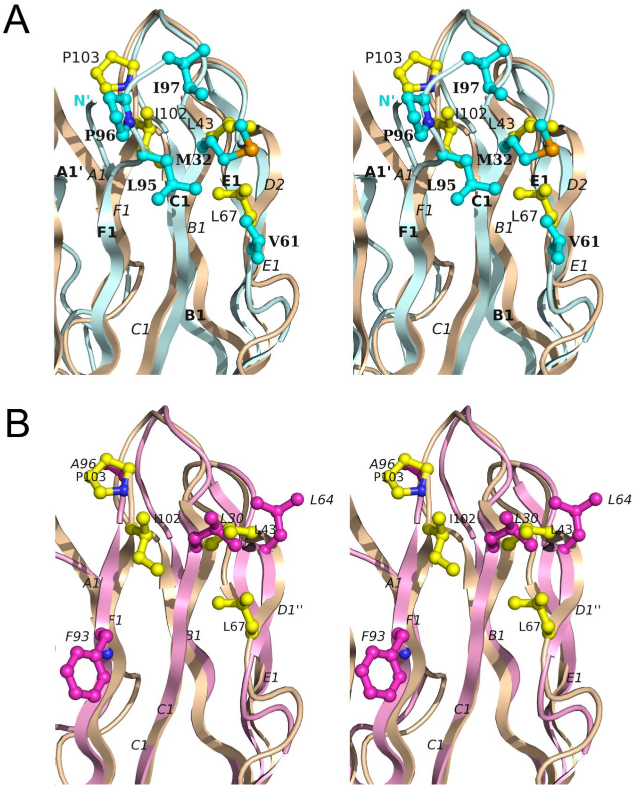

Prediction of usher-binding surface in CsuC and EcpB. N-terminal domain 1 of CsuC and Caf1M (A) and EcpB and Caf1M (B) were superimposed by distance minimization between Cα atoms of corresponding residues. Usher-binding residues in Caf1M (Yu et al., 2012) and hydrophobic surface residues in CsuC and EcpB are shown as balls-and-sticks. CsuC, EcpB, and Caf1M are colored in cyan, magenta, and bronze, respectively. CsuC has large hydrophobic residues in all positions mediating the binding to the usher in classical chaperones. The hydrophobic nature of these positions in archaic chaperones (Figure S2) suggests that archaic and classical systems employ similar mechanisms for the selective binding of pre-assembly complexes to the usher. As CsuC and Caf1M, EcpB possess several hydrophobic residues on the surface of the β-sheet $G_1F_1C_1D_1$. Leu43 in EcpB occupies a hydrophobic position that is conserved in both classical (Met32 in CsuC) and non-classical (Leu43 in Caf1M) chaperones and is implicated in the usher binding in classical chaperones. The hydrophobic position corresponding to Leu67 in Caf1M (Val61 in CsuC) is not present in alternative chaperones. Instead, they contain a conserved hydrophobic position located two residues downstream, which, in EcpB, is occupied by surface exposed Leu79. Hence, alternative chaperones also possess a hydrophobic patch, which they likely use to bind to the usher.
