# Supplementary material for: Structural Insight into Archaic and Alternative Chaperone-Usher Pathways Reveals a Novel Mechanism of Pilus Biogenesis
Source: PLoS Pathog. 2015 Nov 20;11(11):e1005269. doi: 10.1371/journal.ppat.1005269 (PMC4654587; doi:10.1371/journal.ppat.1005269)
Supplement: S11 Fig — Fragments of structures of CsuC, EcpB, and CfaA demonstrate interaction of the subunit C-terminus anchoring arginine with neighbouring residues. CsuC: hydrogen bond between Arg89 and conserved Ser117 in archaic chaperones; EcpB: ionic bond between Arg89 and Glu36; CfaA: ionic bond between Arg90 and conserved Glu48 in CfaA-like chaperones. Residues are shown as balls-and-sticks. (PDF) [file ppat.1005269.s011.pdf]

S11 Fig.

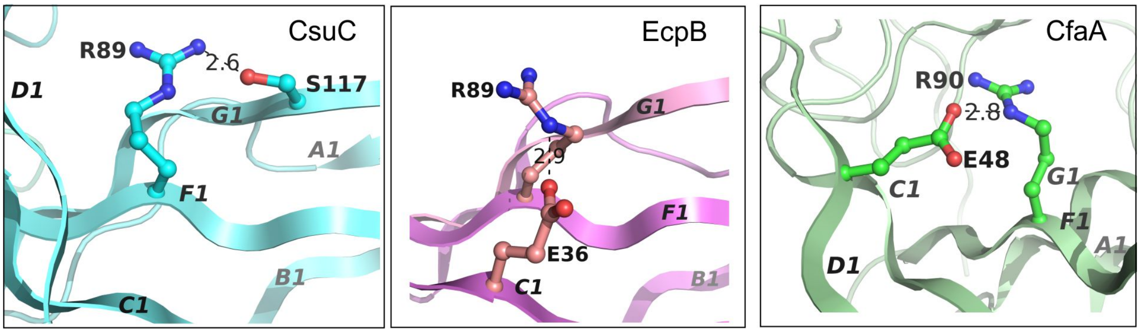

Super-conserved subunit C-terminus-binding arginine in non-classical chaperones is stabilized by an ionic or hydrogen bond to a neighboring residue. Fragments of structures of CsuC, EcpB, and CfaA demonstrate interaction of the subunit C-terminus anchoring arginine with neighboring residues. CsuC: hydrogen bond between Arg89 and conserved Ser117 in archaic chaperones; EcpB: ionic bond between Arg89 and Glu36; CfaA: ionic bond between Arg90 and conserved Glu48 in CfaA-like chaperones. Residues are shown as balls-and-sticks.
