# Supplementary material for: Structural Insight into Archaic and Alternative Chaperone-Usher Pathways Reveals a Novel Mechanism of Pilus Biogenesis
Source: PLoS Pathog. 2015 Nov 20;11(11):e1005269. doi: 10.1371/journal.ppat.1005269 (PMC4654587; doi:10.1371/journal.ppat.1005269)
Supplement: S1 Table — (PDF) [file ppat.1005269.s012.pdf]

S1 Table Diffraction data and refinement statistics

	CsuC:CsuA/B	EcpB	
	(Se)	(Native)	(I)
**Crystal parameters**			
Space group	$P6_422$	$P3_121$	$P3_121$
Cell dimensions	*a*=*b*=94.71, *c*=187.05	*a*=*b*=62.65, *c*=121.14	*a*=*b*=61.76, *c*=120.78
Number of molecules per assymetric unit	1	1	1
**Data collection**			
Beamline	ESRF ID23-1		
Wavelength (Å)	0.979		
Resolution (Å)	49.64-2.40 (2.53-2.40)	54.26-2.40 (2.46-2.40)	60.39-2.62 (2.69-2.62)
Unique observations	20138 (2869)	11265 (805)	8496 (621)
R_{merge}	0.043 (0.539)	0.057 (0.492)	0.127 (0.901)
R_{sym}	0.043 (0.539)	0.057 (0.492)	0.127 (0.901)
$<I>/ \sigma I$	37.4 (6.3)	44.4 (6.6)	31.1 (5.8)
Completeness (%)	99.8 (100)	99.9 (99.7)	99.8 (99.7)
Redundancy	15.18 (16.33)	19.2 (19.4)	37.3 (38.7)
Overall *B* factor from Wilson plot (Å)	54.6	32.3	36.6
**Refinement**			
R_{work}/R_{free} (%)	21.90/26.58	20.5/23.8	-
Number of protein residues	391	185	-
Number of ligands/ions	0	0	-
rmsd stereochemistry			
Bond lengths (Å)	0.009	0.08	-
Bond angles (°)	1.245	1.307	-
Ramachandran analysis (%)*			
Residues in outlier regions	1	0	-
Residues in favoured regions	94.3	98	-
Residues in allowed regions	99	100	-

*Output from Molprobity
