# Supplementary material for: Structural Insight into Archaic and Alternative Chaperone-Usher Pathways Reveals a Novel Mechanism of Pilus Biogenesis
Source: PLoS Pathog. 2015 Nov 20;11(11):e1005269. doi: 10.1371/journal.ppat.1005269 (PMC4654587; doi:10.1371/journal.ppat.1005269)
Supplement: S2 Table — (PDF) [file ppat.1005269.s013.pdf]

S2 Table. Structural comparison of periplasmic chaperones

Compered structures	N Cα	RMSD	Z-score
Domain 1			
CsuC/Caf1M	100	1.9	12.8
CsuC/EcpB	103	1.8	14.2
CsuC/CfaA	111	2.0	15.4
EcpB/Caf1M	93	1.9	12.0
EcpB/CfaA	106	2.5	12.7
CfaA/Caf1M	98	2.3	11.4
Domain 2			
CsuC/Caf1M	70	2.6	5.9
CsuC/EcpB	55	2.5	3.0
CsuC/CfaA	76	3.1	6.3
EcpB/Caf1M	54	2.6	3.1
EcpB/CfaA	64	3.6	5.4
CfaA/Caf1M	72	2.3	8.6
Entire chaperone			
$CsuC_{complex}/Caf1M_{complex}$	172	2.8	14.6
$EcpB_{free}/Caf1M_{complex}$	150	4.3	11.9
$EcpB_{free}/Caf1M_{free}$	145	4.3	11.0
