# Supplementary material for: Structural Insight into Archaic and Alternative Chaperone-Usher Pathways Reveals a Novel Mechanism of Pilus Biogenesis
Source: PLoS Pathog. 2015 Nov 20;11(11):e1005269. doi: 10.1371/journal.ppat.1005269 (PMC4654587; doi:10.1371/journal.ppat.1005269)
Supplement: S3 Table — (PDF) [file ppat.1005269.s014.pdf]

S3 Table. Fraction of structured sequence in pre-assembly complexes

Complex	Structured, %[a]	Accession number (PDB)
Archaic systems		
CsuC-CsuA/B	53[b]	5D6H, this study
Classical systems		
Caf1M-Caf1	100	1P5V
PapD-PapA	99	2UY7
PapD-PapK	100	1PDK
PapD-PapH	100	2J2Z
PapD-PapE	88 (81)[c]	1N0L
FimC-FimH	100	1QUN
FimC-FimA	96	4DWH
FimD-FimC-FimF	90	3BWU
SafB-SafA	100 (89)[d]	2CO6, 2CO7
FaeE-FaeG	91	3GFU

[a] Sequences of N-terminal extensions were excluded from calculations
[b] Sequences with B-factor exceeding 80 Å^2 were considered as unstructured
[c] Two molecules in the asymmetric unit have different degree of folding
[d] Molecules in two different crystals have different degree of folding
