# Supplementary material for: Structural Insight into Archaic and Alternative Chaperone-Usher Pathways Reveals a Novel Mechanism of Pilus Biogenesis
Source: PLoS Pathog. 2015 Nov 20;11(11):e1005269. doi: 10.1371/journal.ppat.1005269 (PMC4654587; doi:10.1371/journal.ppat.1005269)
Supplement: S4 Table — (PDF) [file ppat.1005269.s015.pdf]

S4 Table. Oligonucleotides

Name	Sequence
CsuC-6Hdel-R	GCCTGATGGATCCTCCATTTTG
CsuC-6Hdel-F	TAATGCTAGCGAAAGGGCGAG
6H-CsuAB-R	GTGATGATGAGTAACAGCTGCTTGAGTATTTAC
6H-CsuAB-F	CATCACCATTCAACAGGCTGTACTGTAG
CsuC_R89A-R	CATATGACTGCTCTTTGCCATC
CsuC_R89A-F	CTCTGATTGTAGATGAGTTGCC
CsuC_R174A-R	CCGCAAACTTCTGACCATTATTTTTAAG
CsuC_R89A-F	CTCTTTCGGCACTGAAAACTTC
CsuC_Y196F-R	AGCCAAAAGCAGCCTTTCCTAAAG
CsuC_Y196F-F	TTGTTTTATCAAATAGCACTGTAAAGTTTGC
CsuAB_L10A-R	CTTTAACGTCAACCTGACCAGTAAC
CsuAB_L10A-F	CAAATATCTCAACAGGCTGTACTGTAG
CsuAB_I12A-R	CATTTAATTTAACGTCAACCTGACCAG
CsuAB_I12A-F	CCTCAACAGGCTGTACTGTAGG
CsuAB_AKANA-R	CTTTAGCGTCAACCTGACCAGTAACAGCTG
CsuAB_AKANA-F	CAAATGCCTCAACAGGCTGTACTGTAGGTG
EcpApBAD-F	GAGGAATTAACCATGAAAAAAAAGGTTCTGGCAATAGCTC
EcpApBAD-R	AGCTGCAGATCTCGAGTTAACTGGTCCAGGTAGCGTCG
EcpA_W11A-F	CAGGCTGTAGCGACCGCGTCAGCAACAGCCAA
EcpA_W11A-R	TTGGCTGTTGCTGACGCGGTCGCTACAGCCTG
EcpBpET28-F	AGGAGATATACCATGAAAAAGCACCTTCTGCCTCTCG
EcpBpET28-R	GGTGGTGGTGCTCGAGTTTCACGGGAATGAACTTATCACCC
EcpB_A111G-F	GCGGAAGCAGTGCCCACAGCGCTAC
EcpB_A111G-R	GTAGCGCTGTGGGCACTGCTTCCGC
EcpB_A113G-F	CGATGCGGGCGGAGCCAGTGGCCACAGC
EcpB_A113G-R	GCTGTGGCCACTGGCTCCGCCCGCATCG
EcpB_A115G-F	GTGCCGATGCGGCCGGAAGCAGTGG
EcpB_A115G-R	CCACTGCTTCCGGCCGCATCGGCAC
EcpB_I117G-F	CCAGAATGGTGCCGCCGCGGGCGGAAGCAG
EcpB_I117G-R	CTGCTTCCGCCCGCGGCGGCACCATTCTGG
EcpB_R89A-F	GGCCTGATCAAACCAGACAATCGCGTAGTAGCGCTCTTTTTCATC
EcpB_R89A-R	GATGAAAAAGAGCGCTACTACGCGATTGTCTGGTTTGATCAGGCC
EcpB_R148A-F	CGTAGGCGAGGATCGCCAGCGTCGCATTTC
EcpB_R148A-R	GAAATGCGACGCTGGCGATCCTCGCCTACG
EcpB_Y169F-F	ACTTGCCCGGCATCAGAAAGTAATTCTCTTTACACTCCTTAC
EcpB_Y169F-R	GTAAGGAGTGTAAAGAGAATTACTTTCTGATGCCGGGCAAGT
